# Splice-Switching Antisense Oligonucleotides Targeting Extra- and Intracellular Domains of Epidermal Growth Factor Receptor in Cancer Cells

**DOI:** 10.3390/biomedicines11123299

**Published:** 2023-12-13

**Authors:** Akilandeswari Ashwini Balachandran, Prithi Raguraman, Kamal Rahimizadeh, Rakesh N. Veedu

**Affiliations:** 1 Centre for Molecular Medicine and Innovative Therapeutics, Murdoch University, Murdoch, WA 6150, Australia; 2 Perron Institute for Neurological and Translational Science, Perth, WA 6009, Australia

**Keywords:** epidermal growth factor receptor, splice-switching antisense oligonucleotides, glioblastoma, liver cancer, breast cancer

## Abstract

Cancer is one of the leading causes of death globally. Epidermal growth factor receptor is one of the proteins involved in cancer cell proliferation, differentiation, and invasion. Antisense oligonucleotides are chemical nucleic acids that bind to target messenger ribonucleic acid and modulate its expression. Herein, we demonstrate the efficacy of splice-modulating antisense oligonucleotides to target specific exons in the extracellular (exon 3) and intracellular (exon 18, 21) domains of epidermal growth factor receptor. These antisense oligonucleotides were synthesized as 25mer 2′-O methyl phosphorothioate-modified ribonucleic acids that bind to complementary specific regions in respective exons. We found that PNAT524, PNAT525, PNAT576, and PNAT578 effectively skipped exon 3, exon 18, and exon 21 in glioblastoma, liver cancer, and breast cancer cell lines. PNAT578 treatment also skipped partial exon 19, complete exon 20, and partial exon 21 in addition to complete exon 21 skipping. We also found that a cocktail of PNAT576 and PNAT578 antisense oligonucleotides performed better than their individual counterparts. The migration potential of glioblastoma cancer cells was reduced to a greater extent after treatment with these antisense oligonucleotides. We firmly believe that using these splice-modulating antisense oligonucleotides in combination with existing EGFR-targeted therapies could improve therapeutic outcomes.

## 1. Introduction

Epidermal growth factor receptor (EGFR) is one of the most studied proto-oncogenes involved in several signaling pathways. Deregulation of these signaling mechanisms contributes to cancer initiation, progression, and metastasis. Overexpression of EGFR in cancers is often correlated with poor prognosis, invasiveness, metastasis, and drug resistance [1]. Alternative splicing of *EGFR* yields multiple protein coding and non-coding isoforms. The longest transcript comprises 28 exons, with the exons 1–16 encoding the extracellular domain, exon 17 coding for the transmembrane region, and exons 18–28 encoding the intracellular domain. Exons 2–4 and 8–12 encode the ligand binding domain, and exons 18–24 encode the tyrosine kinase domain [2].

In cancer cells, the two major characteristic alterations of EGFR are EGFR overexpression and mutations activating the tyrosine kinase domain. EGFR overexpression is majorly reported in non-small cell lung cancer (NSCLC) (50–90%), followed by breast cancer (27–90%), liver cancer (68%), and glioblastoma (GBM) (40%). EGFR-activating mutations are reported to be present at 30–40% (east Asian patients) in NSCLC and 30% in GBM [3]. EGFRvIII mutation is characterized by the in-frame deletion of exons 2–7, leading to constitutively active EGFR [4]. This EGFRvIII has been reported to be involved in chemo- and radiotherapy resistance in cancer cells [5]. In glioblastoma, 25–64% of tumors express EGFRvIII and are always associated with EGFR overexpression [6]. In hepatocellular carcinoma, EGFRvIII was detected in 61.8% of samples [7]. EGFRvIII mutations were also seen in 67.8% of primary breast cancers, and 57.1% of infiltrating breast cancers co-expressed wild-type EGFR and EGFRvIII [8]. The most common tyrosine kinase domain mutations are T790M (exon 20), C797S (exon 20), C797X (exon 20), L858R (exon 21), and exon 19 deletion [9,10].

Current therapies approved by the Food and Drug Administration (FDA) for targeting EGFR in cancer include tyrosine kinase inhibitors and monoclonal antibodies. Tyrosine kinase inhibitors such as gefitinib, erlotinib, neratinib, afatinib, dacomitinib, and osimertinib and monoclonal antibodies such as cetuximab, panitumumab, nimotuzumab, and necitumumab are used for treatments in cancers with EGFR dysregulation [11,12,13]. These targeted therapies were reported to be ineffective after a few administrations due to the acquired resistance of cancer cells. The mechanisms involved in acquired resistance include T790M missense mutation and C797S tertiary mutation [10]. In addition, cancer cells also acquired resistance to post-monoclonal antibody therapies via EGFRvIII overexpression, mutations, and activation of downstream signaling pathways; mutations in the extracellular domain; and EGFR heterodimerization [14]. Several other approaches for targeting EGFR were also developed, such as chimeric antigen receptor T cells (CAR-T cells) that target EGFRvIII, antisense oligonucleotides (ASOs), small interfering ribonucleic acid (siRNA), ribozymes, and adjuvant microRNA (miRNA) [15,16,17].

Antisense oligonucleotides are short synthetic single-stranded nucleic acids designed to complement specific regions of the target messenger RNA (mRNA) and modulate target protein expression. These ASOs are of 15 to 30 nucleotides length, bind target mRNA, and can modulate gene expression through different mechanisms as determined by target and ASO chemistry. These mechanisms include RNase-H-mediated mRNA degradation, steric blocking-mediated translational arrest, interaction with untranslated regions, and splice modulation [18]. For RNase-H-mediated activity, ASOs are synthesized as RNA-DNA gapmers since this enzyme specifically recognizes RNA-DNA duplexes and cleaves the RNA strand, thereby degrading the target mRNA [19,20]. The ASOs are synthesized as chemically modified RNA to induce splice-modulating activity. For therapeutic applications, chemical modifications of ASOs are crucial to improve its stability and specificity, and these can be incorporated into the internucleotide linkage, ribose sugar, or nucleobases [21].

The splice-switching ASOs can modify the splicing of precursor-mRNA (pre-mRNA) to induce exon skipping, exon inclusion, or intron retention, thereby modulating the protein expression [20,22]. The motifs that are important for endogenous and induced alternative splicing include branch points, splice sites, polypyrimidine tracts, and exonic and intronic splicing enhancers and silencers [23,24]. The ASOs designed complementary to these regions can prevent the binding of splicing factors, resulting in splice modulation [25]. Modulation of pre-mRNA splicing using ASOs has the potential to treat the underlying cause of diseases that result from aberrant splicing [26] and can bypass or overcome various disease-causing mutations [27].

The therapeutic potential of ASOs is evident with the Food and Drug Adminisration (FDA) and European Medicines Agency (EMA) approval of ten ASOs, as of November 2023, for treating serious diseases. Among these, five ASO drugs induce splice modulation, namely, eteplirsen, nusinersen, golodirsen, viltolarsen, and casimersen [28,29]. The advantage of splice-switching ASOs is that they can be designed to target specific exons of therapeutic importance. Here, we demonstrate the design and evaluation of splice-switching ASOs that target exon 3, exon 18, and exon 21 of *EGFR*. Since complete inactivation or deletion of EGFR results in the failure of organs such as the skin, lung, gastrointestinal tract and affects the formation of neuromuscular synapses and muscle fibers [30,31], splice-switching ASOs are preferred to reduce EGFR activity. Skipping exon 3 leads to a premature stop codon in exon 4, resulting in reduced EGFR expression, and skipping tyrosine kinase domain exons 18 and 21 reduces the tyrosine kinase activity of EGFR. We hypothesize that targeting these exons using splice-modulating ASOs would result in substantial benefits by reducing the expression and tyrosine kinase activity of EGFR.

## 2. Materials and Methods

### 2.1. Antisense Oligonucleotide Design and Synthesis

Antisense oligonucleotides targeting exon 2, exon 3, exon 18, and exon 21 of *EGFR* were designed as 2′-O methyl-modified bases on phosphorothioate backbone (2′-OMe PS)-modified 25mer sequences (Table 1). Scrambled 1 (SCR1) and scrambled 2 (SCR2) sequences were also designed as 2′-OMe PS 25mers that do not anneal to human transcripts (Table 1). All these sequences were synthesized in-house as RNA using GE AKTA Oligopilot 10 DNA/RNA synthesizer (GE Healthcare Life Sciences, Parramatta, NSW, Australia) using solid-phase phosphoramidite chemistry as previously reported [32]. The synthesized oligonucleotides were purified using high-performance liquid chromatography (HPLC from Shimadzu, Sydney, NSW, Australia), characterized by mass spectrometry, and desalted before use.

### 2.2. Cell Lines and ASO Transfection

The human cancer cell lines U87-MG, U251-MG, and Huh-7 were purchased from Cell Bank Australia, and MDA-MB-231 was purchased from the American Type Culture Collection (ATCC). The immortalized human hepatocyte cell line (IHH) was kindly provided by Prof. Grant Ramm’s laboratory at QIMR Berghofer Institute. Normal human fibroblasts (NHF) were kindly provided by the Molecular Therapy Laboratory headed by Prof. Sue Fletcher and Prof. Steve Wilton (Murdoch University Human Ethics Committee approval 2017/101).

The U87-MG and U251-MG cells were grown in 10% Fetal Bovine Serum (FBS) supplemented Eagles Minimal Essential Media (EMEM, ATCC; Cat# 30-2003). Huh-7 and MDA-MB-231 cells were cultured in Dulbecco’s Modified Eagle’s media (DMEM, Thermo Fisher Scientific; Cat# 10569010, Riverstone, NSW, Australia) supplemented with 10% FBS. IHH cells were grown in Dulbecco’s Modified Eagle’s Medium/Nutrient Mixture F-12 Ham (DMEM/F12, Thermo Fisher Scientific; Cat# 10565018, Riverstone, NSW, Australia) media supplemented with 10% FBS, 1% insulin, transferrin, and sodium selenite (ITS, Sigma Aldrich; Cat# I3146-5ML, Castle Hill, NSW, Australia). Normal human fibroblasts were cultured in DMEM supplemented with 1% GlutaMAX and 10% FBS. All cells were maintained in a humidified incubator at 37 °C with 5% CO_2_.

At 80% confluency, the cells were passaged and seeded onto 24-well plates at respective seeding densities in growth media. After the cells reached the desired morphology, they were transfected with ASOs using lipofectamine 3000 (Thermo Fisher Scientific; Cat# L3000015, Riverstone, NSW, Australia) following the manufacturer’s protocol. Initial ASO screening experiments were performed at 50 nM and 400 nM ASO concentrations. The best performing ASOs were shortlisted as PNAT524, PNAT525 for exon 3 skipping, PNAT576 for exon 18 skipping, and PNAT578 for exon 21 skipping. Dose–response experiments were performed for all these ASOs using 2.5 nM, 5 nM, 10 nM, 25 nM, 50 nM, and 100 nM concentrations. The scrambled sequences were transfected at 100 nM concentration. The cells were then incubated for 24 h in a 37 °C incubator with 5% CO_2_.

### 2.3. RNA Extraction and PCR

The cells were collected 24 h post-transfection, lysed using ISOLATE II RNA Mini Kit (Bioline; Cat#: BIO-52073, Eveleigh, NSW, Australia) lysis buffer, and RNA extraction was performed as per the manufacturer’s protocol. The RNA samples were quantified, and 50 ng was used for reverse transcriptase PCR (RT-PCR). Respective primer pairs (Table S1) were used to amplify *EGFR* and *GAPDH* transcripts using the SuperScript III One-Step RT-PCR kit (Thermo Fisher Scientific; Cat# 12574026, Riverstone, NSW, Australia). The PCR conditions and number of cycles (Tables S2–S4) were optimized for all the cell lines used in the study, and respective PCR conditions for the cell line were used for the experiment. The PCR products were then electrophoresed on 2% agarose gel using Tris-acetate-EDTA buffer along with GeneRuler 100 bp DNA Ladder (Thermo Fisher Scientific; Cat# SM0242, Riverstone, NSW, Australia). The gels were stained using Red Safe (iNtRON Biotechnology; Cat# 21141, Burlington, MA, USA) and destained in water for an hour. The images were captured using the Fusion FX gel documentation system (Vilber Lourmat, Marne La Valle, France) and densitometry analysis was carried out using ImageJ 1.52a software.

### 2.4. Sequencing Analysis

To validate specific exon skipping, the full-length and the ASO-induced exon-skipped PCR products were band stabbed as previously described [33]. Using the respective primers, the ‘stabbed products’ were amplified using the AmpliTaq Gold^®^ 360 DNA Polymerase Kit (Thermo Fisher Scientific, Cat# 4398823, Riverstone, NSW, Australia) using their respective primers. The amplified products were verified using 2% agarose gel electrophoresis. The samples were then purified using Diffinity Rapid Tip^®^ (Sigma Aldrich, Cat# D1947-96RXN, Castle Hill, NSW, Australia). The purified products were processed for Sanger sequencing using respective forward and reverse primers at the Australian Genome Research Facility (AGRF), Western Australia.

### 2.5. Western Blotting

The U87-MG and Huh-7 cells were plated in a T25 cm^2^ flask at a seeding density of 5 × 10^5^ cells/flask 24 h before transfection. Then, the cells were transfected with ASO:lipofectamine 3000 complexes at a 100 nM ASO concentration and incubated for 24 h. Post-transfection, the cells were trypsinized, and the pellets were lysed using protein lysis buffer containing 1 M Tris pH 7.5, 5 M NaCl, 0.5 M EDTA pH 8.0, 10% TritonX100, 80% glycerol and protease inhibitor cocktail and incubated on ice for ten minutes. The lysate was then pelleted down at 14,000 rpm for ten minutes. The clear protein lysate was collected and quantified using a Pierce™ BCA Protein Assay Kit (Thermo Scientific, Cat# 23225, Riverstone, NSW, Australia). Protein samples (10 µg) were mixed with Laemmli loading dye and electrophoresed in 10% denaturing polyacrylamide gel at 25 mA using 1X Western blotting running buffer. Once the dye front reached the bottom of the gel, the electrophoresis was stopped, and a wet transfer was performed onto a nitrocellulose membrane (Biorad; Cat # 162-0112, South Granville, NSW, Australia). After 1 h of transfer, the blots were blocked with 5% skimmed milk for an hour. The blots were then washed and probed with anti-EGFR (Abcam, ab52894, Melbourne, VIC, Australia) and anti-GAPDH (Thermo Fisher PA1-988, Riverstone, NSW, Australia) antibodies and incubated overnight at 4 °C. Post-incubation, the blots were washed and incubated with anti-rabbit secondary HRP antibody for two hours at room temperature. After washing, a Clarity Western ECL detection kit (Biorad; Cat# 1705060, South Granville, NSW, Australia) was used to detect the signals. Images were captured using the Fusion FX gel documentation system (Vilber Lourmat, Marne La Valle, France). Densitometry was performed using ImageJ 1.52a software, and the results were normalized first to GAPDH and then to untreated cells. The protein bands were observed to be of the predicted sizes of 175 kDa for EGFR and 37 kDa for GAPDH.

### 2.6. Synergistic Effect of ASOs Targeting the EGFR Tyrosine Domain-Encoding Transcript Sequence

Cells were seeded in 24-well plates, as mentioned above. PNAT576 and PNAT578 were tested for their exon-skipping activity at 100 nM and 200 nM concentrations, and PNAT576 and PNAT578 at 100 nM each in combination, transfected using lipofectamine 3000, as above. After 24 h, the cells were collected, and RNA extraction and RT-PCR were performed.

### 2.7. Migration Assay

The 24-well plates were coated with poly-D-lysine (Merck Millipore; Cat# P7886-50 mg, Bayswater, VIC, Australia) for 1 h before plating. The U251-MG cells were passaged, counted, and seeded at 60,000 cells/well density. After 24 h, the cells were transfected with 100 nM of PNAT524, PNAT525, PANT576, PNAT578, PNAT576+PNAT578, scrambled 2, and untreated using optiMEM medium. After 24 h of transfection, a scratch was made across the well using a 10 µL pipette tip. The existing media was then aspirated completely, EMEM supplemented with 2% FBS was added, and images of the wound were captured at 0 h. Since the 0 h image was captured 24 h after transfection, there was a difference in cell number between ASO-transfected and untreated wells as a consequence of the ASO effect on the cells. Images were also acquired 24 h and 48 h after the scratch was made, and the three timepoint images were analyzed using ImageJ 1.52a software to calculate the scratch area without cells. From this, the percentage wound closure was calculated after 24 h and 48 h with reference to the 0 h image. Three individual sets of experiments (biological replicates) were carried out, and the data shown here are representative of the three experiments.

### 2.8. Statistical Analysis

All experiments were carried out in biological triplicates, and statistical analysis was carried out using GraphPad Prism 8.1.1 software. The data are represented as Mean ± SEM.

## 3. Results

### 3.1. Antisense Oligonucleotide Design and Synthesis

**Figure 1:**
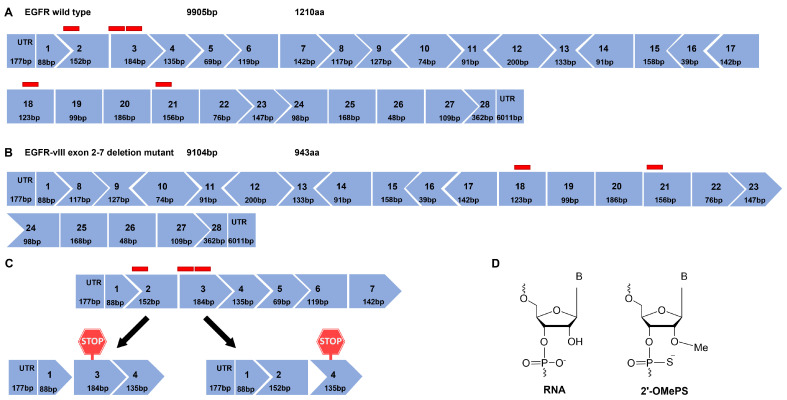
**Exon map of epidermal growth factor receptor (EGFR) and the effect of ASO-induced splice modulation.** (**A**) EGFR wild type exon map and (**B**) EGFR-vIII, an exons 2–7 deletion mutant of EGFR exon map. Red blocks represent the ASO-targeting region of a respective exon. (**C**) An illustration of extracellular domain-targeting ASO-induced exon 2 and exon 3 skipping resulting in a premature stop codon in exon 3 and exon 4, respectively, (**D**) chemical structure of ribonucleic acid (RNA) and 2′ O methyl-modified phosphorothioate RNA.

The exon maps of *EGFR* and *EGFRvIII* are represented in Figure 1A,B, respectively, and exon 2, exon 3, exon 18, and exon 21 were selected as targets for *EGFR* ASO design. The rationale behind choosing these exons, particularly, was that exon 2 or 3 skipping disrupts the reading frame and leads to premature stop codons in the subsequent exon 3 (three stop codons) and exon 4 (one stop codon), respectively (Figure 1C), and either strategy should reduce EGFR protein expression. Since EGFRvIII lacks exons 2–7, ASOs targeting the tyrosine kinase domain were designed. Skipping exon 18 and exon 21 does not alter the reading frame, whereas it might reduce the tyrosine kinase activity of both wild-type and vIII variants of *EGFR*. Exon 18 encodes the region involved in EGFR dimerization, phosphorylation, and activation. Exon 21 encodes the active site of the protein, and both exon 18 and exon 21 include ATP-binding sites [34]. The ASOs were designed using human *EGFR* exon sequences obtained from the Ensembl genome browser, and splicing enhancer regions were predicted using Human Splicing Finder [35]. A graphical representation of the splicing enhancer regions of individual exons is shown in Figure S1. The ASOs were designed complementarily to the regions with higher numbers of splicing enhancer motifs. These ASOs were synthesized in house using GE AKTA with 2′-O methyl phosphorothioate (2′-OMePS) modification and the ASO sequences are listed in Table S1. The structural representation of RNA- and 2′-Ome-modified nucleotide monomers is shown in Figure 1D.

### 3.2. Antisense Oligonucleotide Screening for Splice Modulation of EGFR

**Figure 2:**
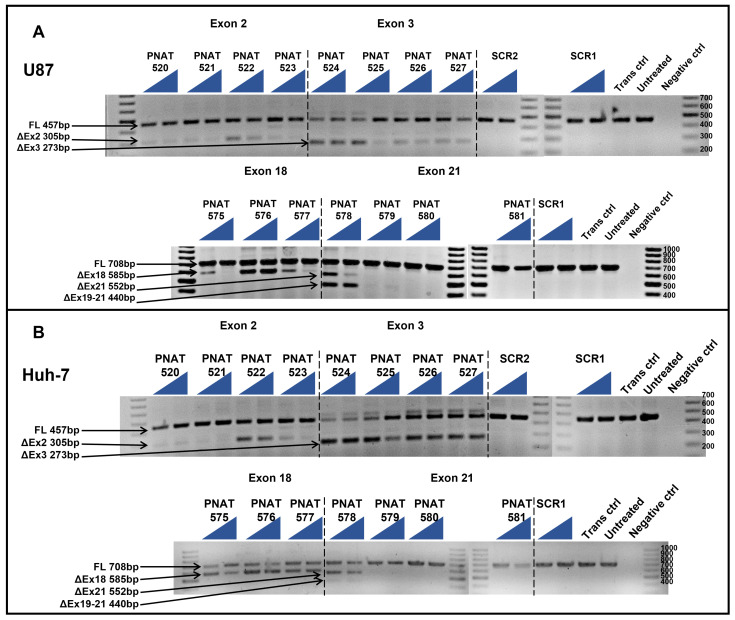
**RT-PCR analysis of initial screen of extracellular and intracellular domain-targeting ASOs.** ASOs targeting exon 2 (PNAT520 to PNAT523), exon 3 (PNAT524 to PNAT527), exon 18 (PNAT575- to PNAT577), exon 21 (PNAT578 to PNAT581), and scrambled sequences SCR1 and SCR2 were transfected at 50 nM and 400 nM concentrations in (**A**) U87-MG cells and (**B**) Huh-7 cells. FL—full length, SCR—scrambled sequence, Trans ctrl—transfection reagent alone control, Negative ctrl—no target PCR control. The triangle represents two concentrations: the lowest (50 nM) and highest (400 nM) concentration of the ASO studied.

The ASOs targeting both extracellular and intracellular domain exons were subjected to initial screening for their exon-skipping activity in U87-MG and Huh-7 cells after transfection using Lipofectamine 3000 at 50 and 400 nM concentrations. PCR amplification across exons 1–4 and agarose gel electrophoresis showed full-length (457 bp), exon 2- (305 bp), and exon 3 (273 bp)-skipped bands in both cell lines (Figure 2A). Similarly, PCR amplification of *EGFR* exon 17–22 and agarose gel electrophoresis showed full-length (708 bp), exon 18- (585 bp), and exon 21 (552 bp)-skipped products in both the cell lines (Figure 2B). This initial screening revealed the best performing ASOs as PNAT524 and PNAT525 for exon 3 skipping, PNAT576 for exon 18, and PNAT578 for exon 21 skipping. The ASOs targeting exon 2 did not show efficient exon-skipping activity when compared to exon 3-targeting ASOs and, hence, were not included in further studies. Surprisingly, exon skipping of PNAT525-induced exon skipping was higher at 50 nM, compared to the 400 nM treatment. In addition to this, two different exon-skipping products were observed when the cells were transfected with PNAT578, which also performed better at 50 nM compared to 400 nM transfection. The scrambled and untreated samples yielded only full-length amplification products.

### 3.3. Sequencing of ASO-Induced EGFR Transcript Products

**Figure 3:**
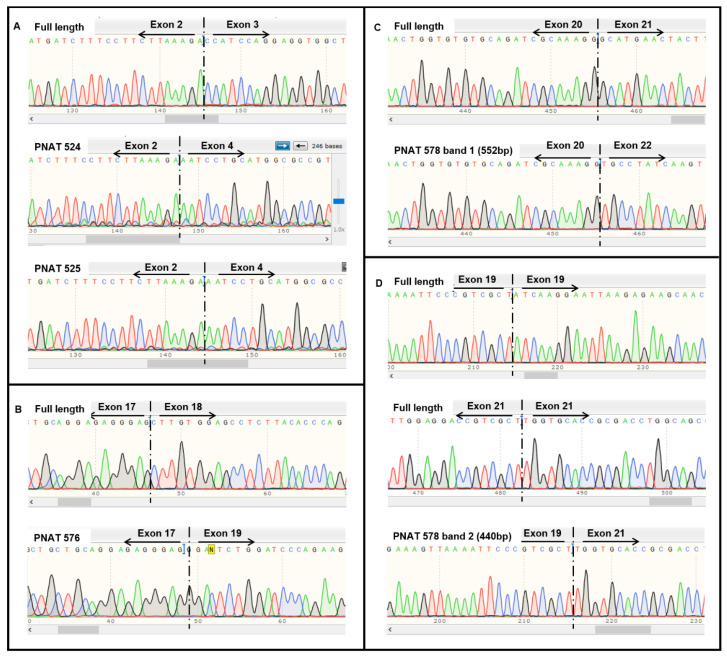
**Sequencing results of extracellular and intracellular domain-targeting ASO-mediated *EGFR* exon skipping.** Sequencing chromatogram of (**A**) full-length, PNAT524, and PNAT 525 exon 3-skipped, (**B**) full-length and PNAT 576 exon 18-skipped, (**C**) full-length and PNAT 578 band 1 exon 21-skipped and (**D**) full-length and PNAT578 band 2 partial exon 19-, complete exon 20-, and partial exon 21-skipped RT-PCR products.

Band-stab sequencing of full-length and exon-skipped products induced by PNAT524, PNAT525, PNAT576, and PNAT578 transfection were analyzed using NCBI BLAST Global align against the *EGFR* full-length sequence. The sequence alignment of exon 3-skipping, exon 18-skipping, and exon 21-skipping products are shown in Figures S2–S4, respectively. The sequencing chromatogram of full-length and exon-skipped bands is shown in Figure 3, confirming that PNAT524 and PNAT525 skipped exon 3 (Figure 3A and Figure S2), PNAT576 skipped exon 18 (Figure 3B and Figure S3), and PNAT578 skipped exon 21 (Figure 3C and Figure S4) completely. In addition to exon 21 skipping, PNAT578 treatment also generated a product resulting from partial skipping of exon 19 (54 bases), complete exon 20 (186 bases) skipping, and partial skipping of exon 21 (28 bases) (Figure 3D and Figure S4). This partial exon 19–21 skipping resulted in an altered reading frame, leading to a premature stop codon in exon 21.

### 3.4. Dose-Dependent ASO-Induced EGFR Exon 3 Skipping

**Figure 4:**
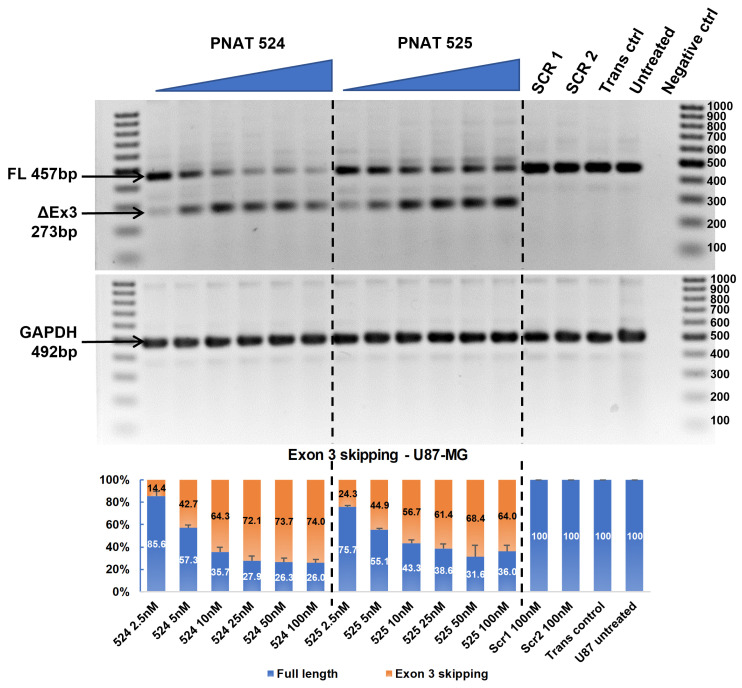
**RT-PCR and densitometry analysis of exon 3 skipping in U87-MG cells.** Agarose gel showing dose-dependent exon 3 skipping following PNAT524 and PNAT525 transfection in U87-MG cells. Densitometry graph of percentage exon 3 skipping is shown. The dose concentrations were 2.5, 5, 10, 25, 50, and 100 nM and the scrambled sequences SCR1 and SCR2 were transfected at 100 nM concentration. The 100 bp ladder was used on either side of the samples and the images represented here were cropped to fit. Densitometry was performed for the biological triplicates and the exon-skipped and full-length bands were normalized to respective GAPDH first and then to the untreated sample. The data are represented as mean ± SEM.

An ASO dose–response study was performed by transfecting 2.5 to 100 nM PNAT524 and PNAT525 in U87-MG, U251-MG, Huh-7, and MDA-MB-231 cell lines. A dose study of these ASOs was performed at 25 nM to 400 nM concentration and no dose-dependent exon-skipping activity was observed beyond 100 nM concentration. It is also evident from Figure 2 that lower concentrations of PNAT525 and PNAT578 induced better exon skipping. Hence, the ASO concentration for the dose study was reduced to 2.5 nM to 100 nM. A good dose-dependent exon 3 skipping was observed in U87-MG cells treated with PNAT524 and PNAT525 until 25 nM concentration. Beyond this concentration, very little increase in exon 3 skipping was seen. The highest concentration of PNAT524 and PNAT525 (100 nM) resulted in 74.0% and 64.0% exon 3 skipping, respectively (Figure 4). Similar results were observed in U251-MG (Figure S5), Huh-7 (Figure S6), and MDA-MB-231 (Figure S7) cells treated with PNAT524 and PNAT525 ASO. It was observed that PNAT524 100 nM induced 75.1%, 72.8%, and 81.0%, and PNAT525 100 nM induced 73.0%, 58.0%, and 68.0% exon 3 skipping in U251-MG, Huh-7, and MDA-MB-231, respectively. The exon-skipping efficiency of these ASOs in non-cancerous cells was studied using IHH and NHF cells. Transfection of PNAT524 and PNAT525 had similar exon-skipping activity in both IHH (Figure S8) and NHF cells (Figure S9). No exon skipping was seen with scrambled transfection and in untreated cells.

### 3.5. Dose-Dependent ASO-Induced EGFR Exon 18 Skipping

**Figure 5:**
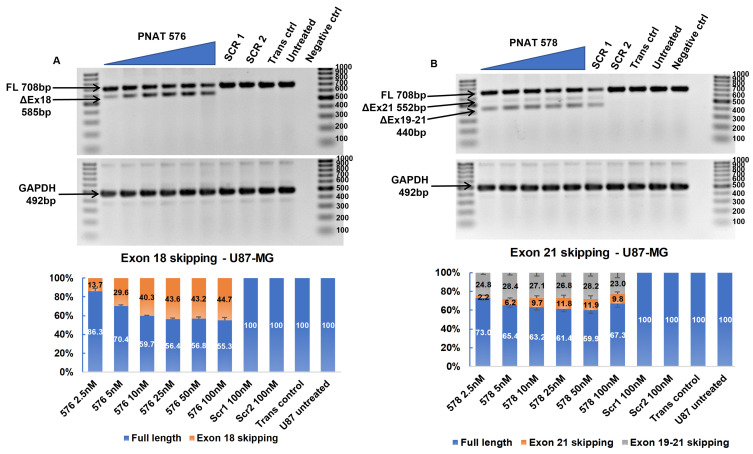
**RT-PCR and densitometry analysis of exon 18 and 21 skipping in U87-MG cells.** (**A**) Agarose gel showing dose-dependent exon 18 skipping following PNAT576 transfection in U87-MG cells and percentage exon 18 skipping assessed by densitometry. (**B**) Agarose gel showing dose-dependent exon 21 skipping following PNAT578 transfection in U87-MG cells and a densitometry graph of percentage exon 21 skipping is shown. The dose concentrations used were 2.5, 5, 10, 25, 50 and 100 nM. The scrambled sequences SCR1 and SCR2 were transfected at 100 nM concentration. The 100 bp ladder was used on either side of the samples and the images represented here were cropped to fit. Densitometry was performed for the biological triplicates and the exon-skipped and full-length bands were normalized to respective GAPDH first and then to the untreated sample. The data are represented as mean ± SEM.

A dose–response study was performed using PNAT576 at 2.5 to 100 nM concentrations in U87-MG, U251-MG, Huh-7, and MDA-MB-231 cells. Dose-dependent exon 18 skipping was observed in U87-MG cells treated with PNAT576 until 25 nM, beyond which the exon skipping plateaued, with 44.7% skipping at the highest (100 nM) concentration (Figure 5A). Similarly, in U251-MG, Huh-7, and MDA-MB-231, exon 18-skipping efficiency after transfection of 100 nM PNAT576 was observed to be 57.5% (Figure S10A), 53.0% (Figure S11A), and 52.6% (Figure S12A), respectively. In addition, PNAT576 treatment in non-cancerous cells showed 57.8% and 58.5% exon 18 skipping in IHH (Figure S13A) and NHF cells (Figure S14A), respectively.

### 3.6. Dose-Dependent ASO-Induced EGFR Exon 21 Skipping

A dose–response study using 2.5 nM to 100 nM concentrations of PNAT578 was performed in U87-MG, U251-MG, Huh-7, and MDA-MB-231 cells. In U87-MG cells treated with PNAT576, 9.8% exon 21 skipping and 23.0% partial exon 19–21 skipping was seen at 100 nM concentration (Figure 5B). Similarly, in U251-MG cells, 5.7% and 22.9% exon 21 and partial exon 19–21 skipping, respectively, was induced at a 100 nM concentration (Figure S10B). In the liver cancer cell line (Huh-7), exon 21 and partial 19–21 skipping was observed to be 34.6% and 16.4%, respectively (Figure S11B). Densitometry analysis showed that dose-dependent multi-exon-skipping activity of PNAT578 was evident in Huh-7 cells. This trend was not seen in MDA-MB-231 triple-negative breast cancer cells. When transfected with PNAT578, MDA-MB-231 cells showed minimal exon 21 skipping (3.6%) and increased partial exon 19–21 skipping (30%) (Figure S12B). In the case of IHH cells, exon 21 skipping was 20.9% and partial skipping was 16.9% (Figure S13B). Likewise, in NHF, 9.2% exon 21 skipping and 24.9% partial skipping were observed (Figure S14B). The extent of complete exon 21 skipping and partial exon 19–21 skipping differs in different cell lines.

### 3.7. Synergistic Effect of Tyrosine Kinase Domain-Targeting ASOs

**Figure 6:**
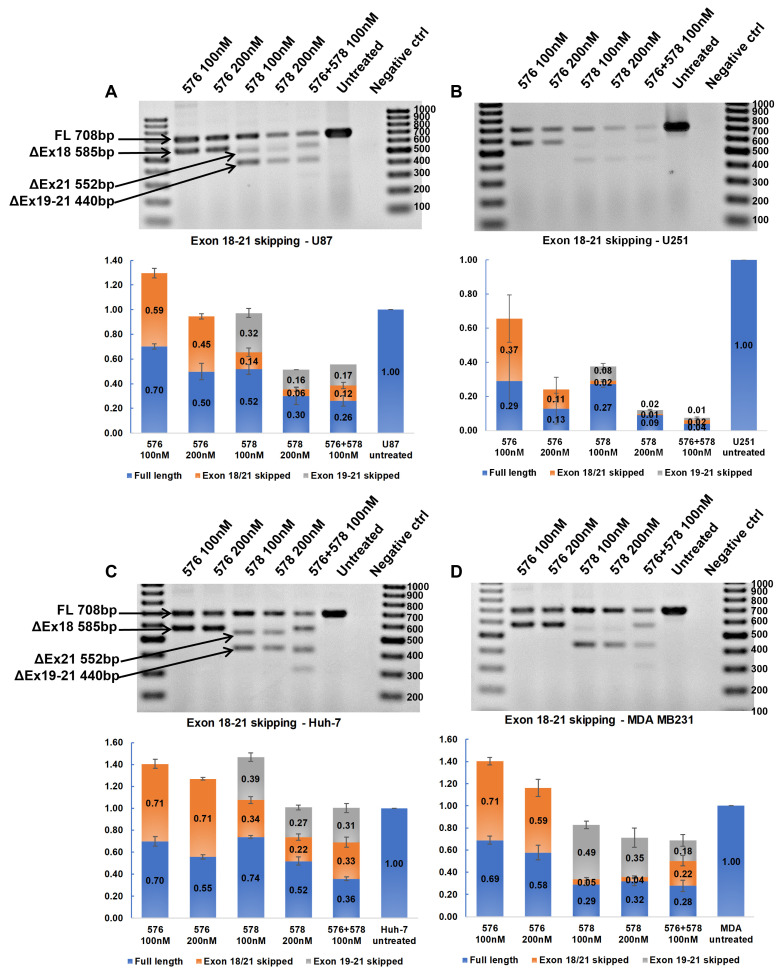
**RT-PCR analysis to assess synergistic effects of tyrosine kinase domain-targeting ASOs.** Agarose gel showing RT-PCR products representing skipping of exons 18–21 induced by PNAT576, PNAT578, and PNAT576+578 in combination in (**A**) U87-MG, (**B**) U251-MG, (**C**) Huh-7, and (**D**) MDA-MB-231 cells. The 100 bp ladder was used on either side of the samples and the images represented here were cropped to fit. Densitometry was performed for the biological triplicates and the exon-skipped and full-length bands were normalized to respective GAPDH first and then to the untreated sample. The data are represented as mean ± SEM.

The tyrosine kinase domain-targeting ASOs were transfected in combination to determine if dual-targeting ASOs could have a synergistic effect. U87-MG, U251-MG, Huh-7, and MDA-MB-231 cells were transfected individually with 100 nM and 200 nM of PNAT576 and PNAT578 and in combination with PNAT576 and PNAT578 each at 100 nM concentration. In U87-MG cells, dose-dependent exon 18–21 skipping was induced in cells treated with individual and ASO combinations (Figure 6A). These treatments were even more effective in U251-MG cells with an almost 90% reduction in *EGFR* transcript expression by using PNAT576 and PNAT578 combination (Figure 6B). In contrast, the expression level of the *EGFR* transcript was not affected by the combination treatment in Huh-7 cells, but there was an increase in the percentage of exon 18–21 skipping with increasing ASO concentration (Figure 6C). In the case of MDA-MB-231, a dose-dependent exon 18–21-skipping trend was seen with a moderate reduction in the transcript levels (Figure 6D). The combination of PNAT576 and PNAT578 induced multiple transcripts involving complete excision of exons 18, 20, and 21, as well as a spliceoform retaining portions of exons 19 and 21, an out-of-frame transcript. This combination treatment successfully removes exons that are important for the tyrosine kinase activity of EGFR.

### 3.8. Effect of Exon-Skipping ASOs on EGFR Protein Expression

**Figure 7:**
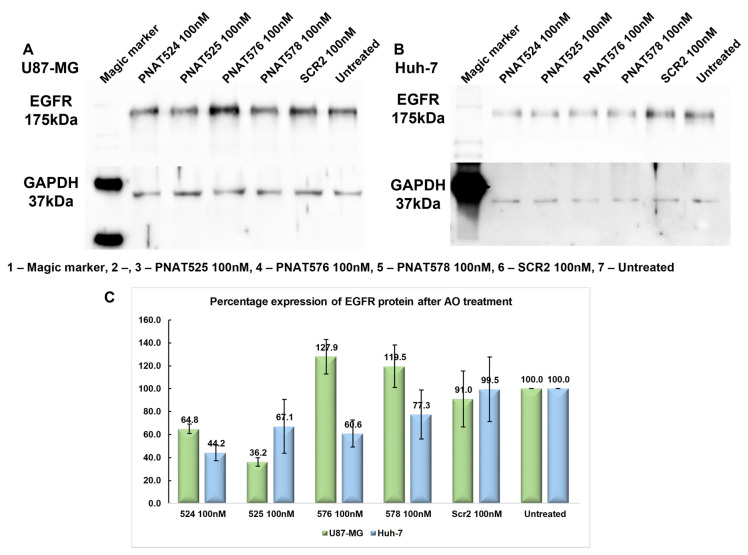
**Effect of exon-skipping ASO treatment on EGFR protein expression.** (**A**) Western blotting of protein lysates from PNAT524-, PNAT525-, PNAT576-, and PNAT578-transfected U87-MG cells, probed with anti-EGFR and anti-GAPDH antibodies. (**B**) Western blotting of protein lysates from PNAT524-, PNAT525-, PNAT576-, and PNAT578-transfected Huh-7 cells, probed with anti-EGFR and anti-GAPDH antibodies. (**C**) Densitometry analysis of EGFR protein expression after ASO treatment in U87-MG and Huh-7 cells. The biological triplicate data are represented as mean ± SEM.

The effectiveness of the exon-skipping ASOs in reducing EGFR protein expression was assessed by Western blotting. For this purpose, U87-MG and Huh-7 cells were transfected with a 100 nM concentration of ASOs PNAT524, PNAT525, PNAT576, PNAT578, and a scrambled control SCR2 and incubated for 24 h. Total protein (10 µg) was electrophoresed and immune-blotted using anti-EGFR and anti-GAPDH antibodies, and anti-rabbit secondary antibody labelled with HRP. The EGFR and GAPDH band intensities for U87-MG ASO-treated samples (Figure 7A) and Huh-7 ASO-treated samples (Figure 7B) were quantified by densitometry. The obtained values for EGFR bands were normalized first against GAPDH and then by the values derived from the untreated samples, represented in Figure 7C.

The extracellular domain-targeting ASOs PNAT524 and PNAT525 treatment resulted in 64.8% and 36.2% of EGFR protein expression, respectively, in U87-MG cells when compared to that in the untreated cells. Surprisingly, the tyrosine kinase domain-targeting ASOs PNAT576 and PNAT578 treatment increased EGFR expression to 127.9% and 119.5%, respectively, which was unexpected right at the beginning (Figure 7A,C). In Huh-7 cells, the EGFR protein expression after PNAT524 and PNAT525 ASO treatment was 44.2% and 67.1%, respectively. Similarly, PNAT576 and PNAT578 treatment resulted in 60.6% and 77.3% of EGFR expression when compared with untreated cells (Figure 7B,C). The extracellular domain-targeting ASO treatment reduced EGFR protein expression in both Huh-7 and U87-MG cancer cells. Since the anti-EGFR antibody binds to the extracellular region of EGFR, protein alterations as a result of exon 18 and 21 skipping would not be reflected by this analysis.

### 3.9. Effect of Exon-Skipping ASOs on Cancer Cell Migration

**Figure 8:**
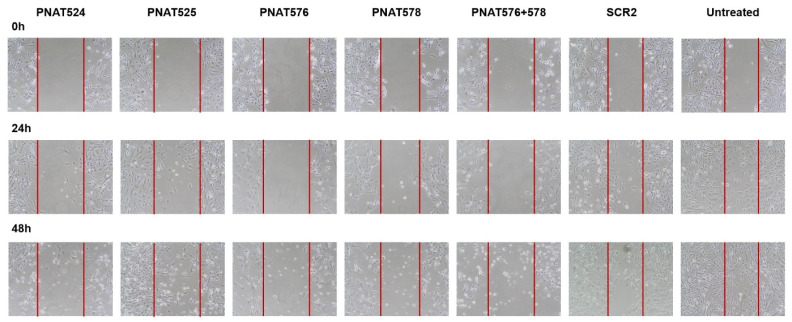
**Effect of exon-skipping ASO treatment on cancer cell migration.** U251-MG cells were treated with PNAT524, PNAT525, PNAT576, PNAT578, and PNAT576+578 combination, and after 24 h of transfection, a wound was made. Images were captured at 0 h, 24 h, and 48 h for all the treatments. After 48 h, the wound was mostly closed in untreated and scrambled SCR2-treated cells compared to ASO-treated cells.

The effect of *EGFR* exon-skipping ASOs on the migrating potential of cancer cells was studied using a wound healing assay [36]. A wound was made in U251-MG monolayer cell cultures transfected with PNAT524, PNAT525, PNAT576, PNAT578, PNAT576+578 combination, and scrambled control SCR2 at 100 nM. Images were captured at 0 h, 24 h, and 48 h after a wound was created and the images of PNAT524-, PNAT525-, PNAT576-, PNAT578-, and PNAT576+578 combination-treated cells compared to the baseline (0 h) images (Figure 8). Since the cells were transfected with ASO for 24 h prior to creating the wound, the cell density appears to be different between the untreated and the ASO-treated 0 h images.

**Figure 9:**
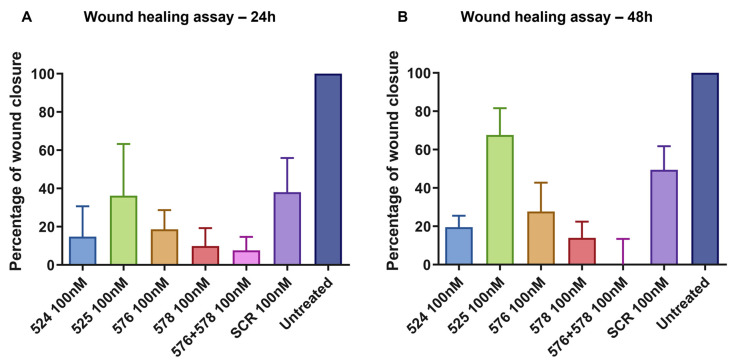
**Relative wound closure, expressed as a proportion, relative to untreated control.** A comparison of wound closure after (**A**) 24 h and (**B**) 48 h in U251-MG cells transfected with PNAT524, PNAT525, PNAT576, PNAT578, PNAT576+PNAT578, and SCR2 ASOs. Data of the biological triplicates are represented as mean ± SEM.

The area of the wound without cells were quantified using ImageJ 1.52a software and a relative wound closure percentage was obtained. After 24 h, PNAT576+578 reduced the migration of U251-MG cells better compared to other ASO treatments (Figure 9A). It was observed that after 48 h, PNAT524, PNAT576, PNAT578 and PNAT576+578 combination treatments reduced the migration potential of U251-MG cells when compared to scrambled ASO (Figure 9B). The PNAT576+578 ASO combination reduced the migration potential of U251-MG cells drastically. However, more dead cells were observed in these ASO-transfected wells when compared to scrambled SCR2 and untreated control wells.

## 4. Discussion

EGFR is frequently dysregulated in cancer cells by over-expression or activating mutations. EGFR-targeted therapies such as monoclonal antibodies and tyrosine kinase inhibitors are currently available therapeutics for EGFR-driven cancers. Several other molecules targeting EGFR were also developed and tested for efficacy [37]. Cancer cells gain resistance towards these EGFR-targeted therapies, usually through acquired mutations in the tyrosine kinase domain. The tyrosine kinase inhibitors of EGFR have been improved to overcome the acquired resistance of previous tyrosine kinase inhibitors; however, resistance towards these therapies continues to evolve [9,10].

Antisense oligonucleotides can be designed to regulate mRNA expression by RNase H-mediated cleavage, steric blocking [38], or splice modulation [18]. These ASOs can be modified chemically to improve their stability and efficacy [39,40,41,42,43]. Modulating the expression of *EGFR* using splice-switching ASOs provides strategies to overcome drug resistance caused by EGFR over-expression and acquired mutations. The EGFR-targeting ASOs studied so far were designed to target the translation-initiation or termination regions of *EGFR*, leading to translation blockade, and were reported to have inhibitory effects in different cancer cells [44,45,46,47,48,49]. The advantage of splice-modulating ASOs is that they can be designed to target specific exons of *EGFR*, particularly those necessary for tyrosine kinase activity and signaling.

When this study was designed and performed, no reports existed on splice-modulating *EGFR*-specific ASOs. Recently, a study was published focusing on the effect of splice-modulating *EGFR*-specific ASOs targeting exon 16, 18, and 21 on NSCLC cells [50]. The ASOs reported were designed to target the 3′ and 5′ splice junction of exons and are morpholino ASOs, whereas the ASOs evaluated here were designed within the exon and also at the exon–intron junction and are 2′-O-methyl PS modified. Morpholino oligonucleotides are synthesized by complete replacement of the phosphodiester backbone linkage of the ASOs [51]. The 2′-O-methyl PS modifications are generated by replacing the 2′ hydroxyl group of sugar moiety with a methyl group [52]. The previous study generated ASOs targeting the tyrosine kinase domain of EGFR, whereas this study is focused on both the extracellular domain and the tyrosine kinase domain. This study successfully demonstrated the scope and exon-skipping activity of ASOs targeting extracellular and intracellular domains of EGFR. These ASOs were designed to target exons 3, 18, and 21 and were found to be effective in inducing splice-modulation in glioblastoma (U87-MG, U251-MG), liver (Huh-7), and breast (MDA-MB-231) cancer cell lines.

The splice-modulating ASOs PNAT524, PNAT525, and PNAT576 induced single exon skipping, whereas PNAT578 induced multiple skipping. Amongst the two ASO candidates shortlisted for exon 3 skipping, PNAT524 performed better. We observed that exon 3 skipping induced by PNAT524 was greater than 70% in all the cancer cell lines at 100 nM concentration. But the exon-skipping efficiency of PNAT524 reached a plateau after 25 nM concentration. This could be attributed to an ineffective delivery of ASOs into the cells by the transfection reagent. To overcome this issue, self-internalizing ASOs could be used as alternatives for transfection reagents. It was noted that exon 18 skipping by PNAT576 was over 44%, and that PNAT578-mediated exon 21 skipping was only ~28%. The of percentage exon 18 skipping shown by Madanayake et al. at a 1 µM concentration of the Ex18 ASO was very minimal when compared to 44% of exon 18 skipping by PNAT576 at a 100 nM concentration. In addition, no exon 21 skipping was observed even at a 5 µM concentration of the Ex21 ASO treatment [50]. But PNAT578 induced 28% exon 21 skipping and the PNAT576+PNAT578 combination treatment skipped exon 21 to a greater extent. This shows that PNAT576 and PNAT578 have better splice modulating potential.

PNAT578-mediated multi-exon skipping patterns were also observed in the MDA-MB-231 breast cancer cell line. In glioblastoma (U87-MG, U251-MG) and liver cancer (Huh-7) cells, PNAT578 induced both exon 21 and exon 19–21 multiple skipping, while in breast cancer (MDA-MB-231) cells, exon 19–21 multiple skipping predominated, and minimal exon 21 skipping was seen occasionally. This suggests that the ASOs modulate splicing in a similar manner in different cancer cell types, but the extent of exon skipping varies. We also demonstrated that the PNAT576+PNAT578 combination skipped exons 18–21 (up to 74% and 99% of the transcript) in U87-MG and U251-MG glioblastoma cells, respectively, and 64% and 72% in Huh-7 and MDA-MB-231 cells, respectively. By skipping exon 18, exon 20, and exon 21 together using PNAT576 and PNAT578 in combination, the ATP-binding site (exon 18) and both the ATP-binding and active site (exon 21) of EGFR can be effectively removed. In addition, we tested these ASOs in non-cancerous cells, and similar exon-skipping patterns were observed due to the expression of EGFR in most cells, as it is vital for cell proliferation. It was also observed that EGFR protein levels were reduced when U87-MG and Huh-7 cells were transfected with exon 3-skipping ASOs (PNAT524 and PNAT525). This aligns with the altered reading frame resulting from exon 3 skipping, leading to a premature stop codon in exon 4, thereby reducing EGFR protein expression.

Our results revealed that the migration of U251-MG cells was considerably reduced when treated with PNAT524, PNAT576, PNAT578, and PNAT576+PNAT578 in combination. The migration potential of U251-MG glioblastoma cells was hampered upon these ASO treatments compared to the scrambled SCR2 treatment. We tried to study the effect of these ASOs on migration in other cancer cell lines (U87-MG, Huh-7, and MDA-MB-231). Since this experiment was carried out for 72 h in reduced serum media, U87-MG, Huh-7, and MDA-MB-231 cells lost their morphology, which was not feasible to measure the wound-closure percentage.

Acquired resistance to EGFR-targeting tyrosine kinase inhibitor is attributed to both EGFR-dependent and EGFR-independent mechanisms. Depending on the inhibitor used, the occurrence of several activating mutations such as T790M, C797X, L858R, G719X, and L861Q leads to therapy resistance. These activating mutations were reported to occur in exons 18–21 [53,54]. Sueangoen et al. reported seven activating mutations in exons 19–23 of EGFR in hepatocellular carcinoma tissue that all lead to erlotinib resistance [55]. Since the tyrosine kinase domain is prone to mutations post-treatment, the ASOs studied here could have a therapeutic impact and combination therapies could potentially reduce the invasive characteristics of cancer cells [56].

## 5. Conclusions

EGFR is one of the most studied oncogenic targets, and therapies specific to EGFR are continually evolving. Cancer cells also attempt to escape these therapies by acquiring resistance via amplification and mutations, with tyrosine kinase domain exons 18–24 frequently acquiring mutations post-treatment. Hence, removing mutation-prone exons using splice-modulating ASOs may help overcome these acquired resistances. We demonstrated that splice-modulating ASOs effectively skipped exons 3, 18, 20, and 21 from *EGFR* mRNA in vitro. The results were consistent in the different cancer cell lines tested and showed dose-dependent exon-skipping activity. Since transfection of ASOs is not feasible in vivo, we are currently working on different conjugation strategies for transfection reagent-free internalization of these ASOs in cancer cells. It is speculated that skipping exons 3, 18, 20, and 21 using splice-modulating ASOs could be of therapeutic benefit. Further work is needed to understand the potential of these ASOs in reducing drug resistance and improving therapeutic outcomes.

## Figures and Tables

**Table 1:** Sequence of antisense oligonucleotides targeting *EGFR* and scrambled oligonucleotides.

PNAT No.	ASO Name	Sequence 5′–3′
**Exon 2**		
PNAT520	EGFR 1H2A (+08+32)	CUG CGU GAG CUU GUU ACU CGU GCC U
PNAT521	EGFR 1H2A (+33+58)	GAA AAU GAU CUU CAA AAG UGC CCA A
PNAT522	EGFR 1H2A (+65+89)	CUC ACA GUU AUU GAA CAU CCU CUG G
PNAT523	EGFR 1H2A (+111+135)	CAU AAU UCC UCU GCA CAU AGG UAA U
**Exon 3**		
PNAT524	EGFR 1H3A (−04+21)	ACC AGC CAC CUC CUG GAU GGU CUA A
PNAT525	EGFR 1H3A (+25+49)	CCA CUG UGU UGA GGG CAA UGA GGA C
PNAT526	EGFR 1H3A (+60+84)	UCU GAU GAU CUG CAG GUU UUC CAA A
PNAT527	EGFR 1H3A (+141+165)	CAG CUC CUU CAG UCC GGU UUU AUU U
PNAT528	EGFR 1H3A (+156+180)	UAA AUU UCU CAU GGG CAG CUC CUU C
**Exon 18**		
PNAT575	EGFR H18A (+16+40)	GGU UGG GAG CUU CUC CAC UGG GUG U
PNAT576	EGFR H18A (+50+74)	AAU UCA GUU UCC UUC AAG AUC CUC A
PNAT577	EGFR H18A (+90+114)	CGU GCC GAA CGC ACC GGA GCC CAG C
**Exon 21**		
PNAT578	EGFR H21A (+7+31)	CCA AGC GAC GGU CCU CCA AGU AGU U
PNAT579	EGFR H21A (+40+64)	CCA GUA CGU UCC UGG CUG CCA GGU C
PNAT580	EGFR H21A (+114+138)	GUA UUC UUU CUC UUC CGC ACC CAG C
PNAT581	EGFR H21A (+132+156)	UUU GCC UCC UUC UGC AUG GUA UUC U
SCR1		UCA UCG AUG GCA GCU GCG UGU CGU U
SCR2		UCA CCU GAG CGG AGG GGA CCU GUG G

## Data Availability

Data are contained within the article and Supplementary Materials.

## Supplementary Materials

The following supporting information can be downloaded at: https://www.mdpi.com/article/10.3390/biomedicines11123299/s1.
