# Supplementary material for: Splice-Switching Antisense Oligonucleotides Targeting Extra- and Intracellular Domains of Epidermal Growth Factor Receptor in Cancer Cells
Source: Biomedicines. 2023 Dec 13;11(12):3299. doi: 10.3390/biomedicines11123299 (PMC10741442; doi:10.3390/biomedicines11123299)
Supplement: Supplementary file 1 [file biomedicines-11-03299-s001.zip › biomedicines-2702943-supplementary.pdf]

Novel splice-switching antisense oligonucleotides targeting extra and intracellular domains of epidermal growth factor receptor in cancer cells

Supplementary materials

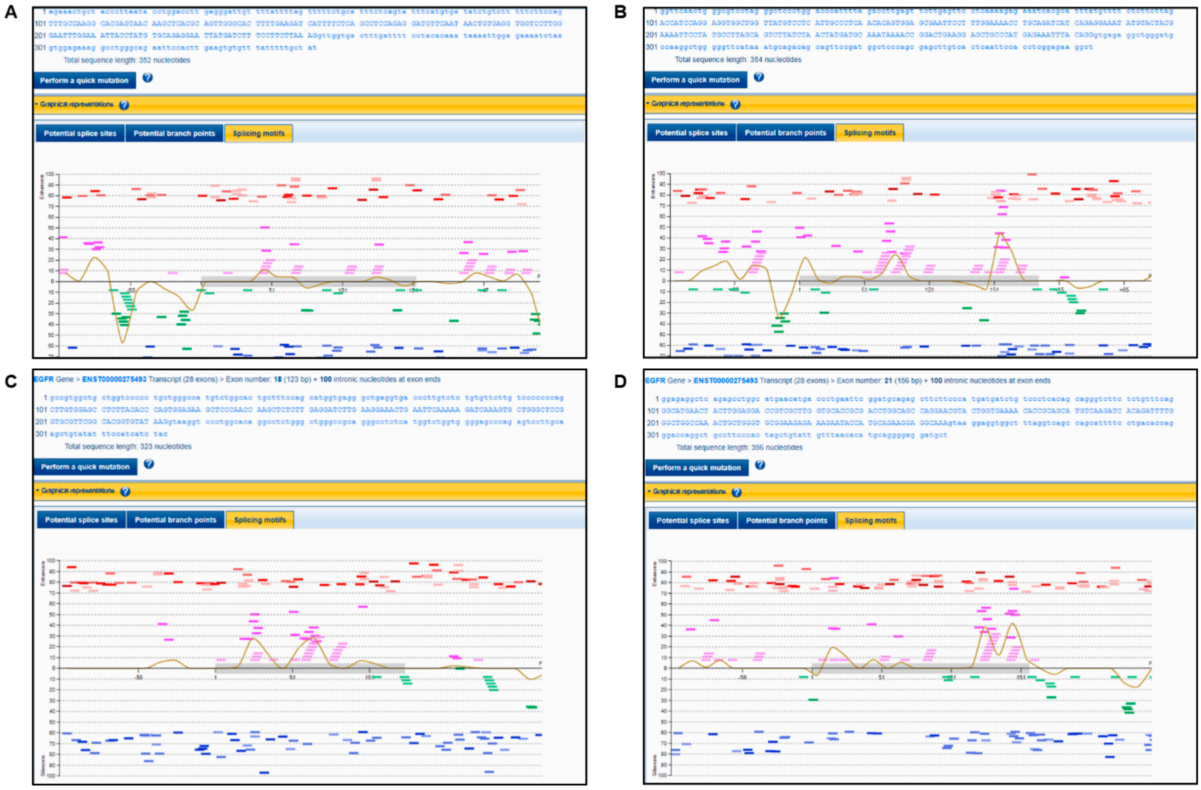

Figure S1: Graphical representation of the splicing enhancer regions of *EGFR*. Splicing enhancer regions of (A) exon 2, (B) exon 3, (C) exon 18, and (D) exon 21 of *EGFR* as observed in human splicing finder. The complete sequence of the respective exon, along with 100 nucleotides of the intronic region on both ends, was used for this analysis. The ASOs were designed complementary to the regions with increased splicing enhancer motifs.

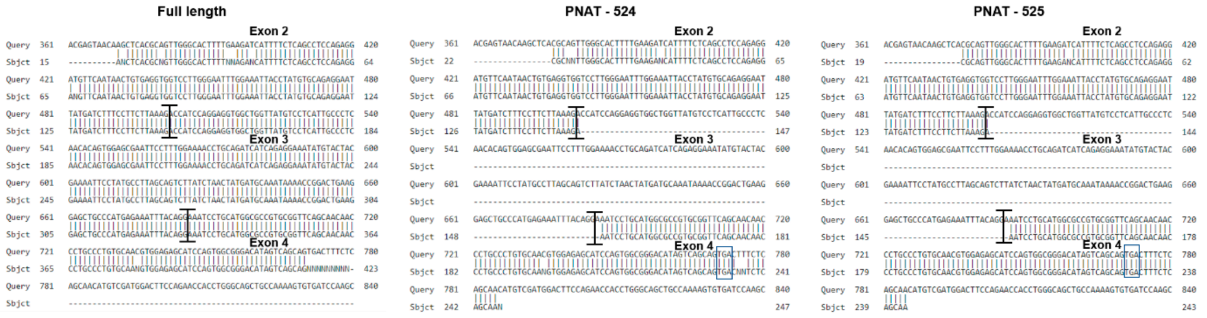

Figure S2: BLAST alignment of exon 3 skipping sequencing results. The sequence obtained for full length, PNAT524 skipped, and PNAT525 skipped was aligned globally with the sequence of EGFR. Exons are marked, and PNAT524 and PNAT525 clearly demonstrate exon 3 skipping. The premature stop codon is marked in PNAT524 and PNAT525 alignment, which is a result of the altered reading frame due to exon 3 skipping.

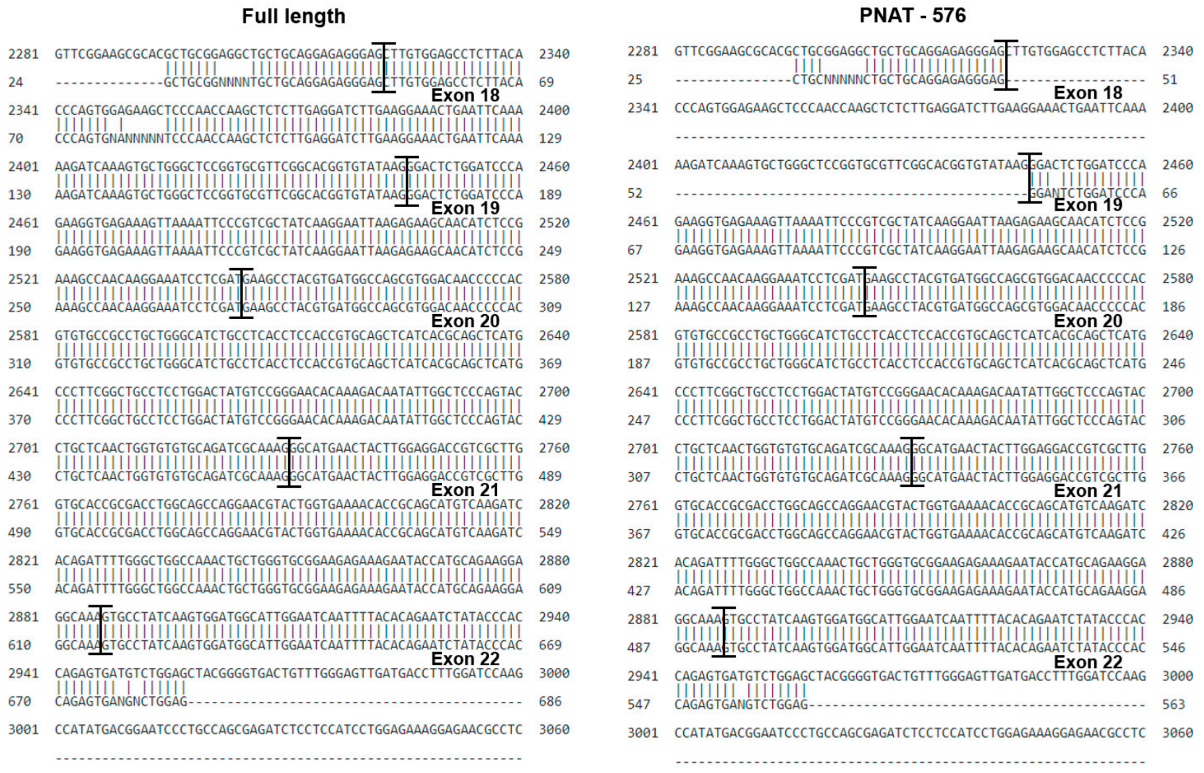

Figure S3: BLAST alignment of exon 18 skipping sequencing results. The sequence obtained for full length and PNAT576 skipped was aligned globally with the sequence of EGFR. Exons are marked, and PNAT576 clearly demonstrates exon 18 skipping.

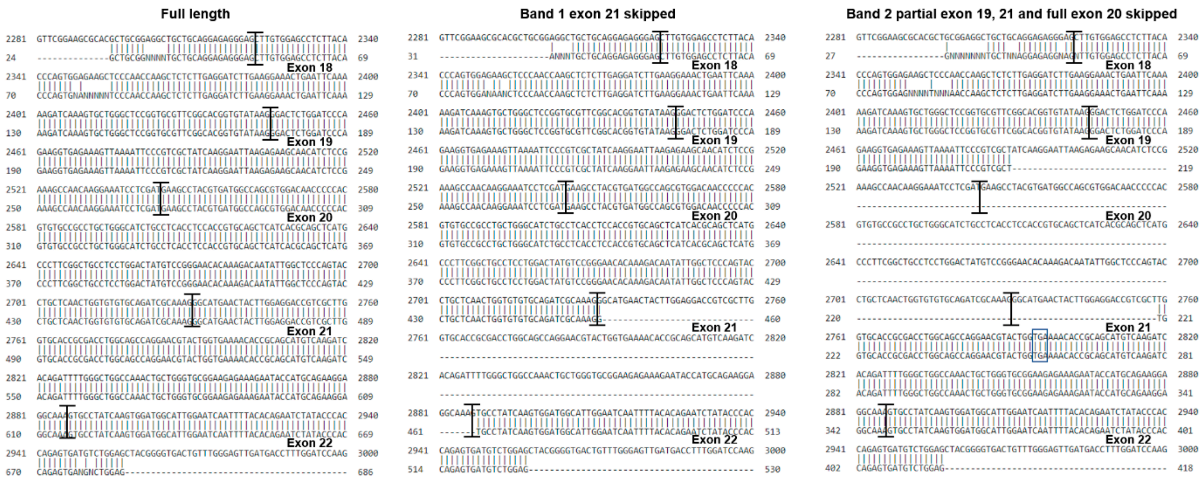

Figure S4: BLAST alignment of exon 21 skipping sequencing results. The sequence obtained for full length and band 1 and 2 of PNAT578 skipping was aligned globally with the sequence of EGFR. Exons are marked, and band 1 of PNAT578 clearly demonstrates exon 21 skipping. Whereas band 2 of PNAT578 shows partial skipping of exon 19, complete exon 20 skipping, and partial skipping of exon 21. This results in an altered reading frame resulting in a premature stop codon in exon 21 (marked).

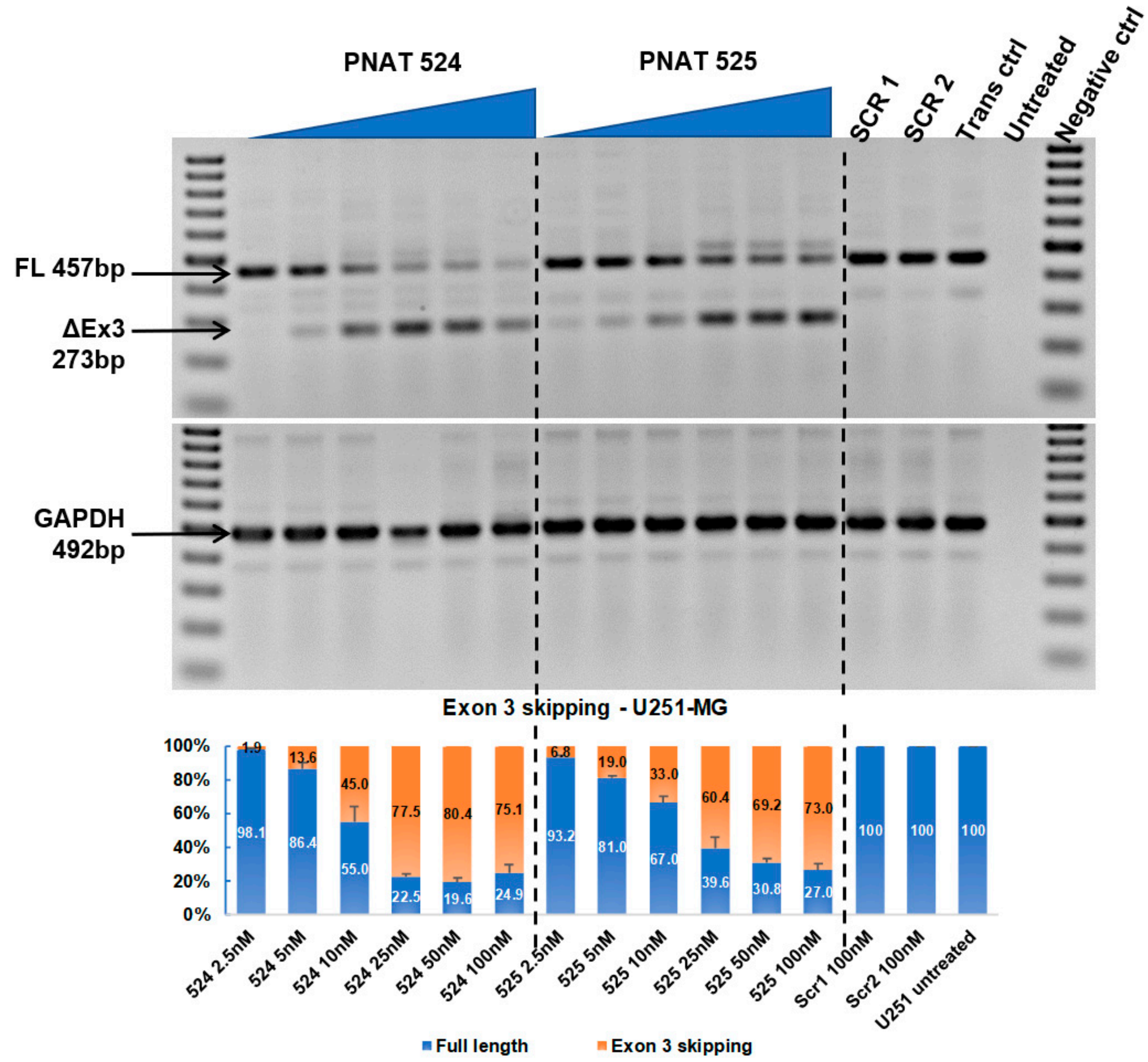

Figure S5: RT-PCR and densitometry analysis of exon 3 skipping in U251-MG cells. Agarose gel showing dose-dependent exon 3 skipping following PNAT524 and PNAT525 transfection in U251-MG cells and densitometry graph of percentage exon 3 skipping. The dose concentration was 2.5, 5, 10, 25, 50 and 100nM. The scrambled sequences SCR1 and

SCR2 were transfected at 100nM concentration. Densitometry was performed for the biological triplicates and the exon skipped and full-length bands were normalised to respective GAPDH first and then to untreated sample. The data are represented as mean±SEM.

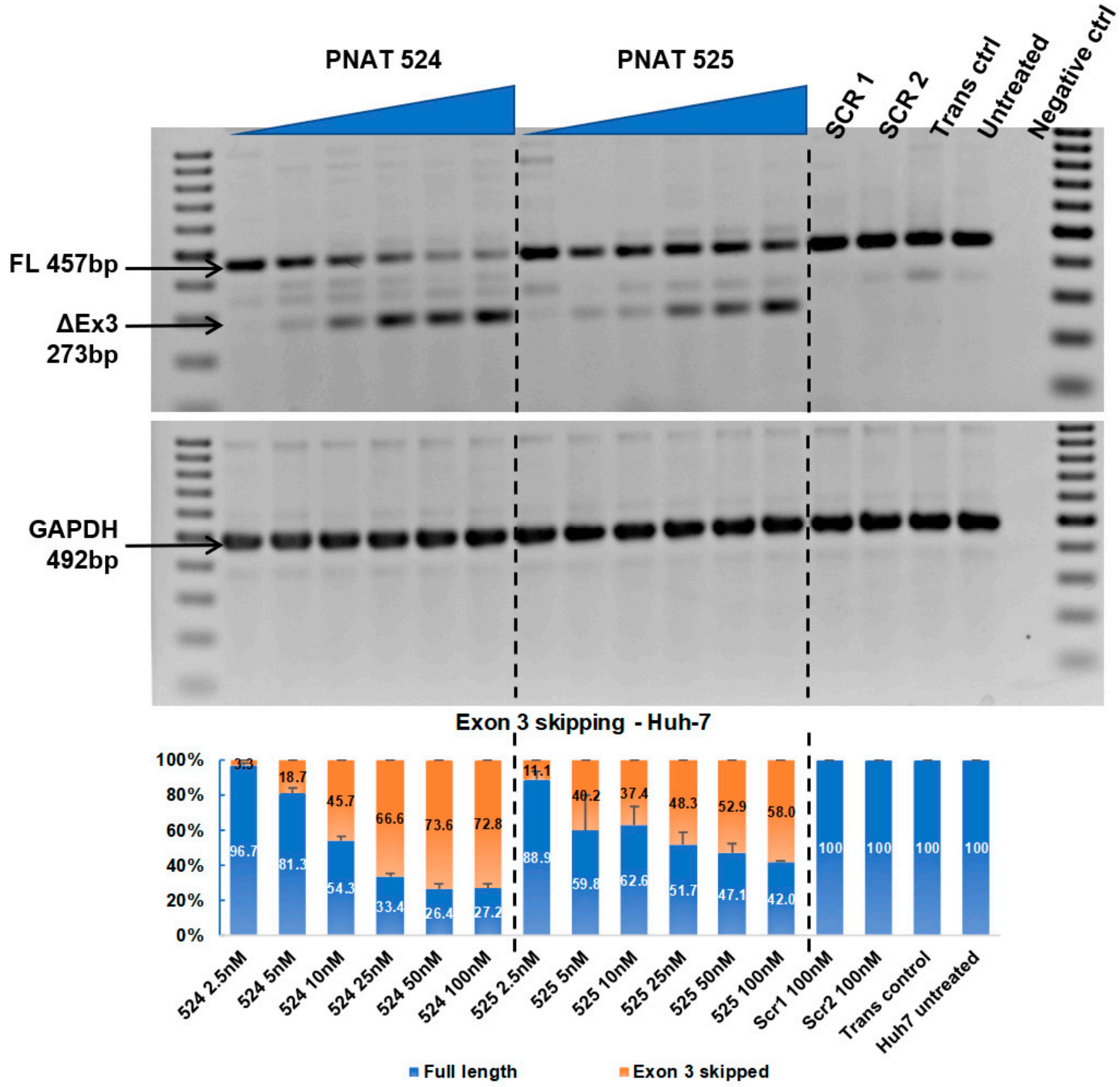

Figure S6: RT-PCR and densitometry analysis of exon 3 skipping in Huh-7 cells. Agarose gel showing dose-dependent exon 3 skipping following PNAT524 and PNAT525 transfection in Huh-7 cells and densitometry graph of percentage exon 3 skipping. The dose concentration was 2.5, 5, 10, 25, 50 and 100nM. The scrambled sequences SCR1 and SCR2 were transfected at 100nM concentration. Densitometry was performed for the biological triplicates and the exon skipped and full-length bands were normalised to respective GAPDH first and then to untreated sample. The data are represented as mean±SEM.

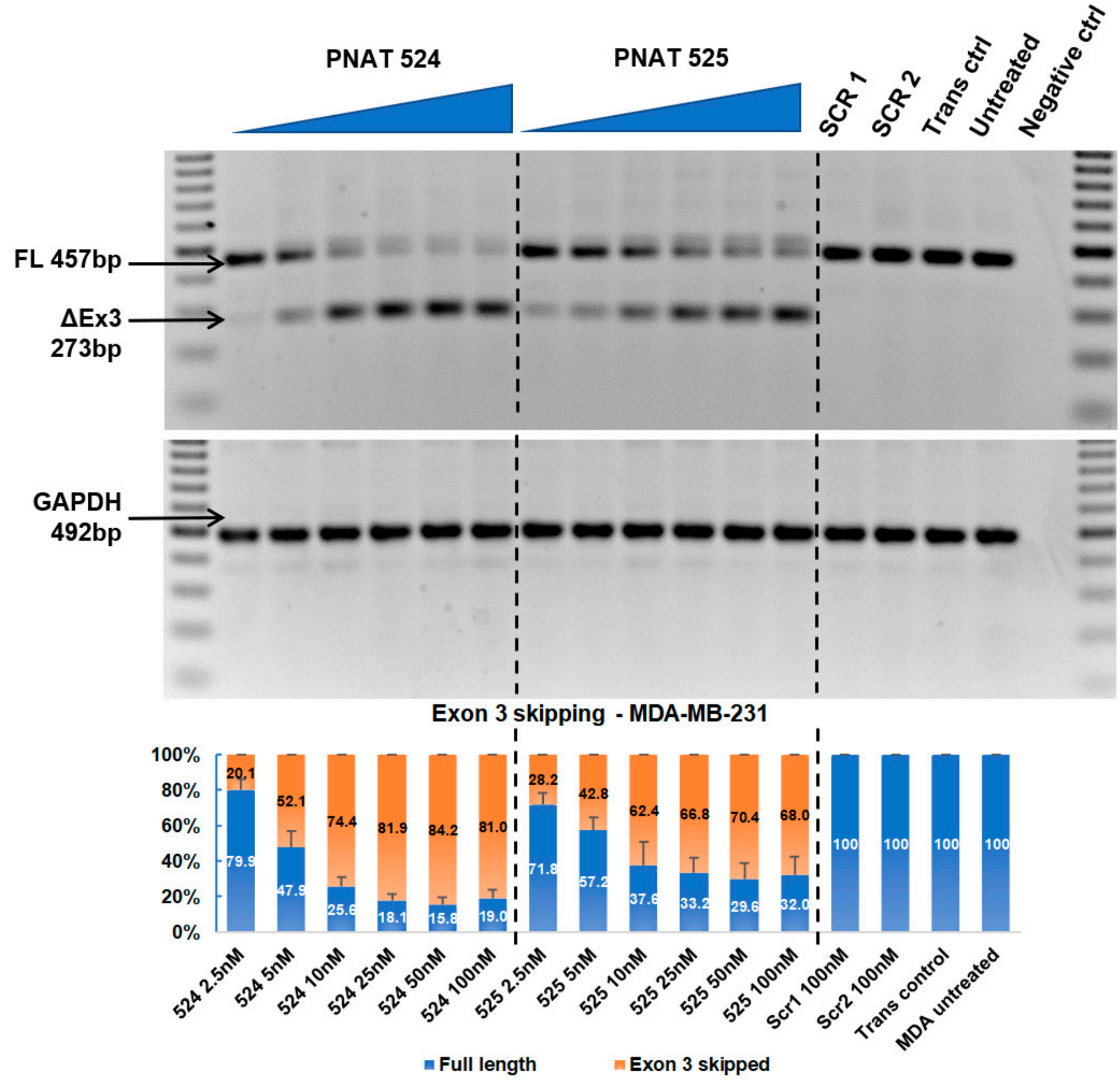

Figure S7: RT-PCR and densitometry analysis of exon 3 skipping in MDA-MB-231 cells. Agarose gel showing dose-dependent exon 3 skipping following PNAT524 and PNAT525 transfection in MDA-MB-231 cells and densitometry graph of percentage exon 3 skipping. The dose concentration was 2.5, 5, 10, 25, 50 and 100nM. The scrambled sequences SCR1 and SCR2 were transfected at 100nM concentration. Densitometry was performed for the biological triplicates and the exon skipped and full-length bands were normalised to respective GAPDH first and then to untreated sample. The data are represented as mean±SEM.

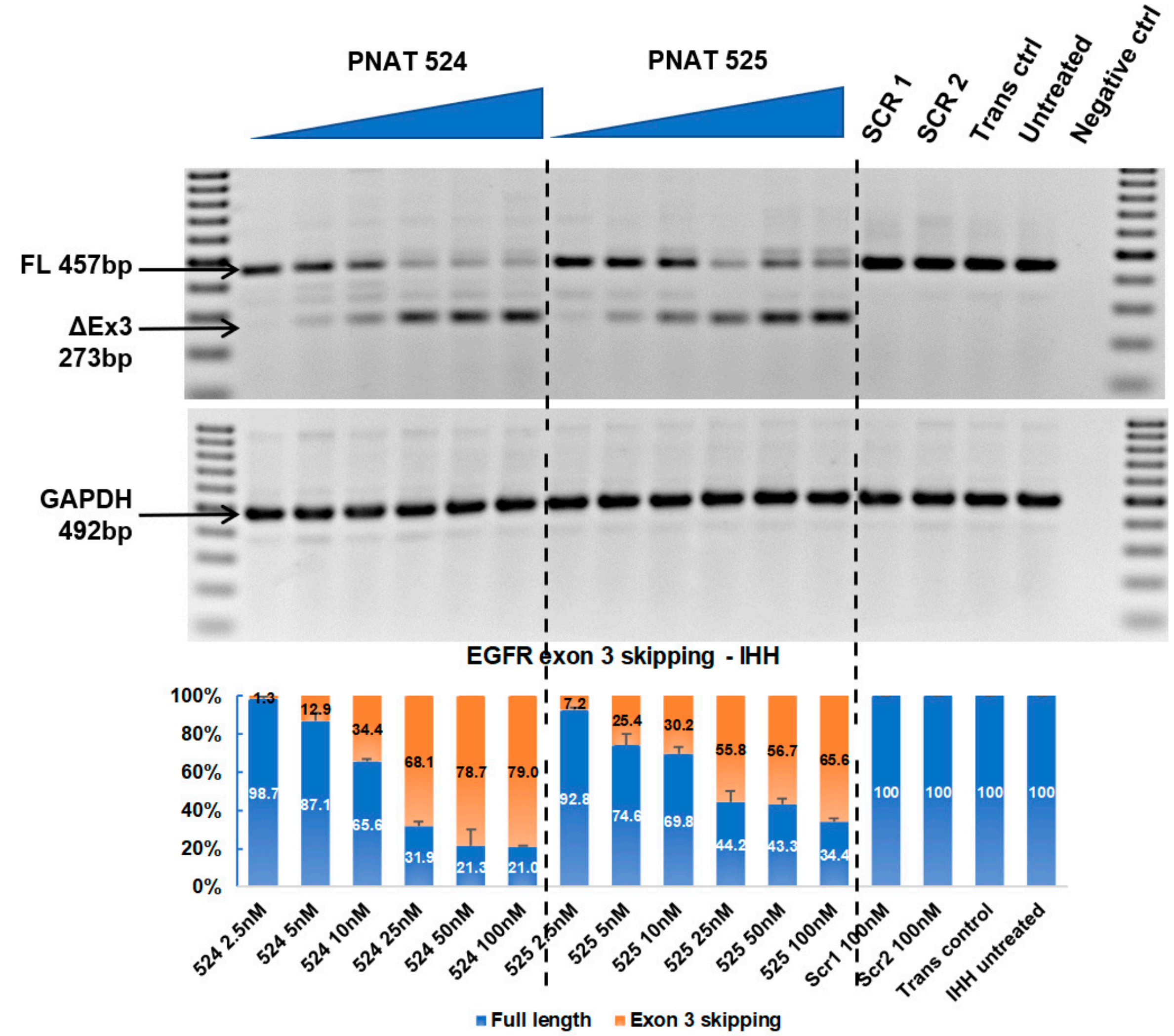

Figure S8: RT-PCR and densitometry analysis of exon 3 skipping in IHH cells. Agarose gel showing dose-dependent exon 3 skipping following PNAT524 and PNAT525 transfection in IHH cells and densitometry graph of percentage exon 3 skipping. The dose concentration was 2.5, 5, 10, 25, 50 and 100nM. The scrambled sequences SCR1 and SCR2 were transfected at 100nM concentration. Densitometry was performed for the biological triplicates and the exon skipped and full-length bands were normalised to respective GAPDH first and then to untreated sample. The data are represented as mean±SEM.

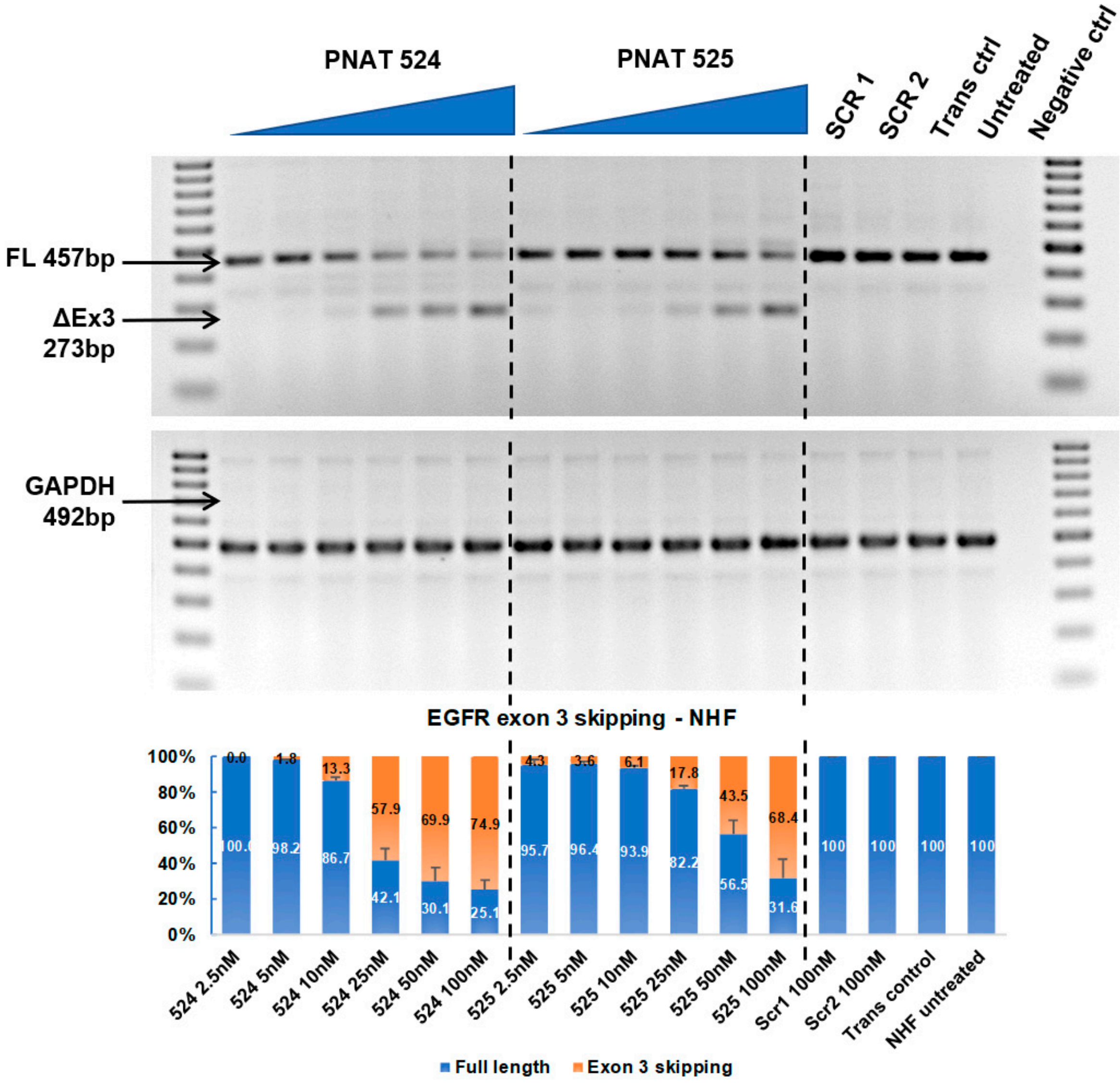

Figure S9: RT-PCR and densitometry analysis of exon 3 skipping in NHF cells. Agarose gel showing dose-dependent exon 3 skipping following PNAT524 and PNAT525 transfection in NHF cells and densitometry graph of percentage exon 3 skipping. The dose concentration was 2.5, 5, 10, 25, 50 and 100nM. The scrambled sequences SCR1 and SCR2 were transfected at 100nM concentration. Densitometry was performed for the biological triplicates and the exon skipped and full-length bands were normalised to respective GAPDH first and then to untreated sample. The data are represented as mean±SEM.

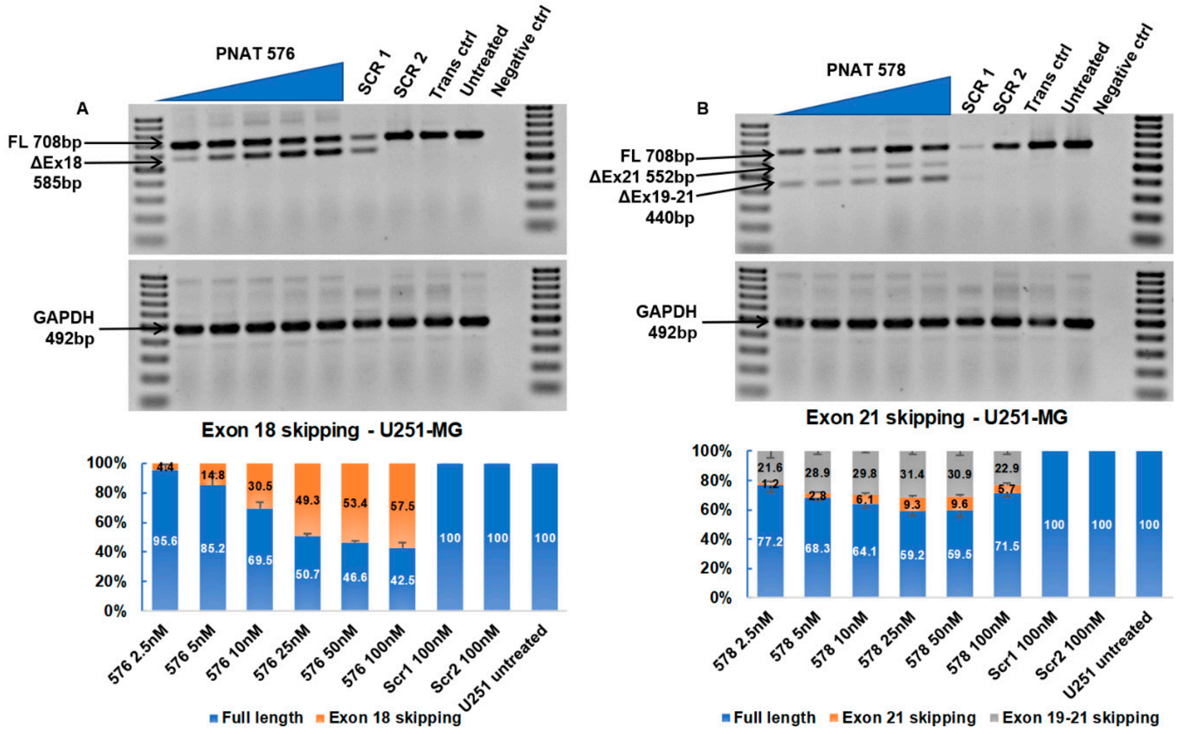

Figure S10: RT-PCR and densitometry analysis of exon 18 and 21 skipping in U251-MG cells. (A) Agarose gel showing dose-dependent exon 18 skipping following PNAT576 transfection in U251-MG cells and percentage exon 18 skipping assessed by densitometry (B) Agarose gel showing dose-dependent exon 21 and partial 19-21 skipping following PNAT578 transfection in U251-MG cells and percentage exon 21 and partial 19-21 skipping assessed by densitometry. The dose concentration was 2.5, 5, 10, 25, 50 and 100nM. The scrambled sequences SCR1 and SCR2 were transfected at 100nM concentration. Densitometry was performed for the biological triplicates and the exon skipped and full-length bands were normalised to respective GAPDH first and then to untreated sample. The data are represented as mean±SEM.

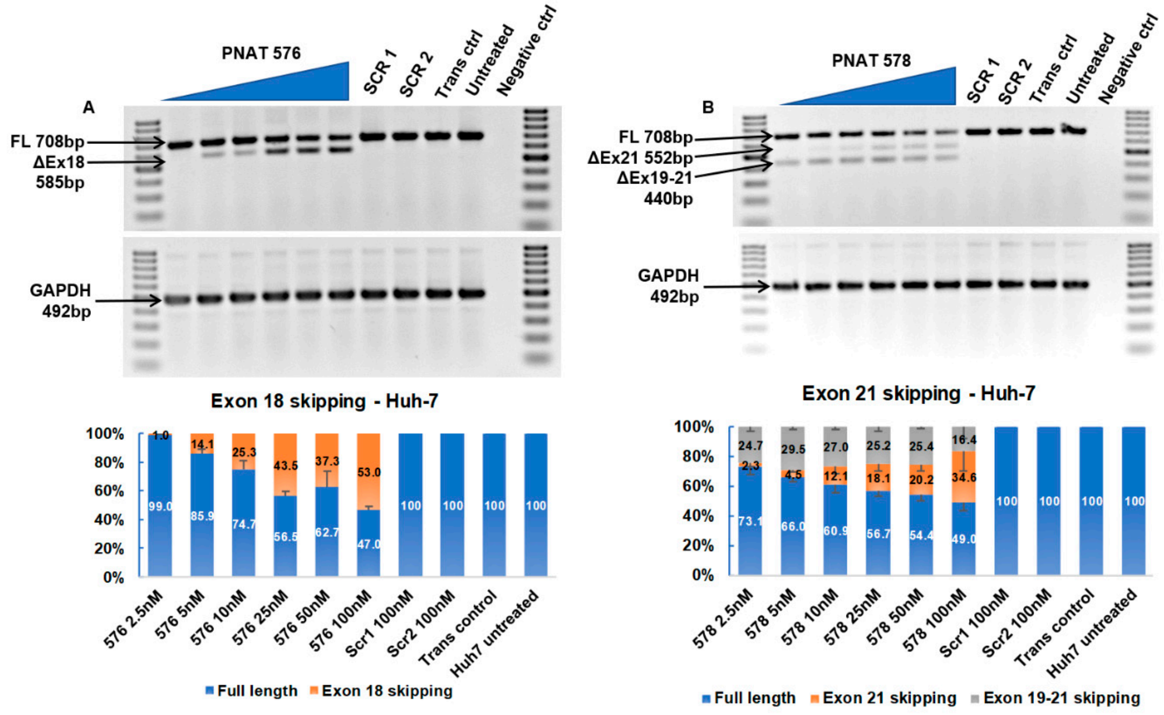

Figure S11: RT-PCR and densitometry analysis of exon 18 and 21 skipping in Huh-7 cells. (A) Agarose gel showing dose-dependent exon 18 skipping following PNAT576 transfection in Huh-7 cells and percentage exon 18 skipping assessed by densitometry (B) Agarose gel showing dose-dependent exon 21 and partial 19-21 skipping following PNAT578 transfection in Huh-7 cells and percentage exon 21 and partial 19-21 skipping assessed by densitometry. The dose concentration was 2.5, 5, 10, 25, 50 and 100nM. The scrambled sequences SCR1 and SCR2 were transfected at 100nM concentration. Densitometry was performed for the biological triplicates and the exon skipped and full-length bands were normalised to respective GAPDH first and then to untreated sample. The data are represented as mean±SEM.

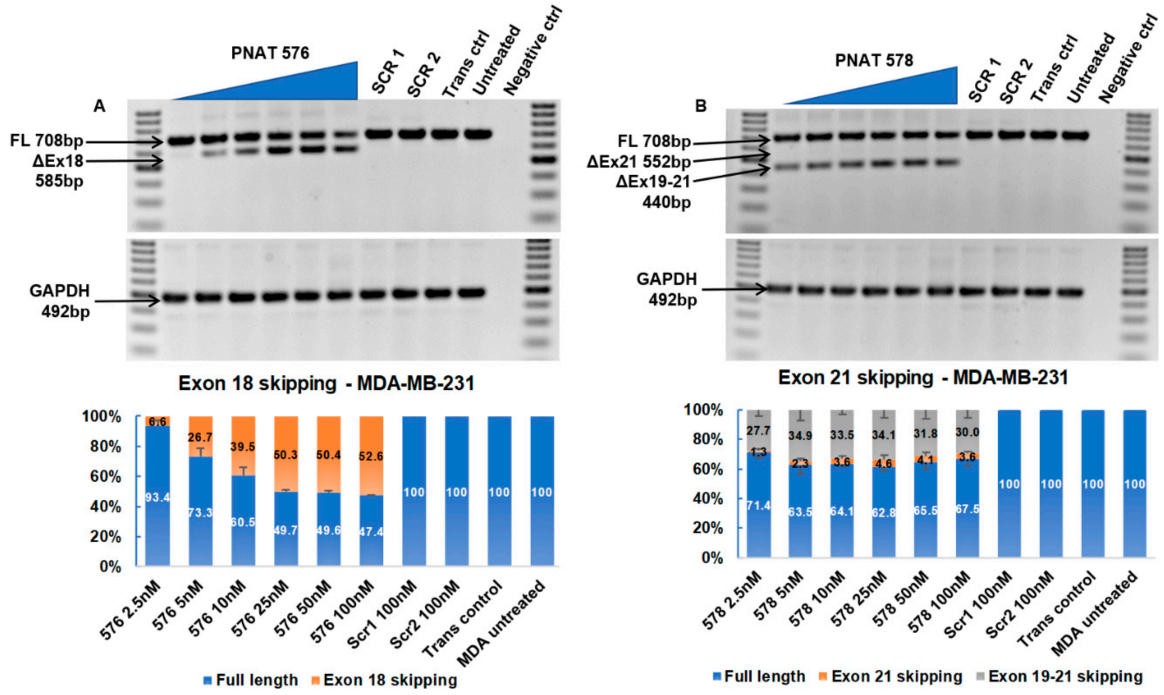

Figure S12: RT-PCR and densitometry analysis of exon 18 and 21 skipping in MDA-MB-231 cells. (A) Agarose gel showing dose-dependent exon 18 skipping following PNAT576 transfection in MDA-MB-231 cells and percentage exon 18 skipping assessed by densitometry (B) Agarose gel showing dose-dependent exon 21 and partial 19-21 skipping following PNAT578 transfection in MDA-MB-231 cells and percentage exon 21 and partial 19-21 skipping assessed by densitometry. The dose concentration was 2.5, 5, 10, 25, 50 and 100nM. The scrambled sequences SCR1 and SCR2 were transfected at 100nM concentration. Densitometry was performed for the biological triplicates and the exon skipped and full-length bands were normalised to respective GAPDH first and then to untreated sample. The data are represented as mean±SEM.

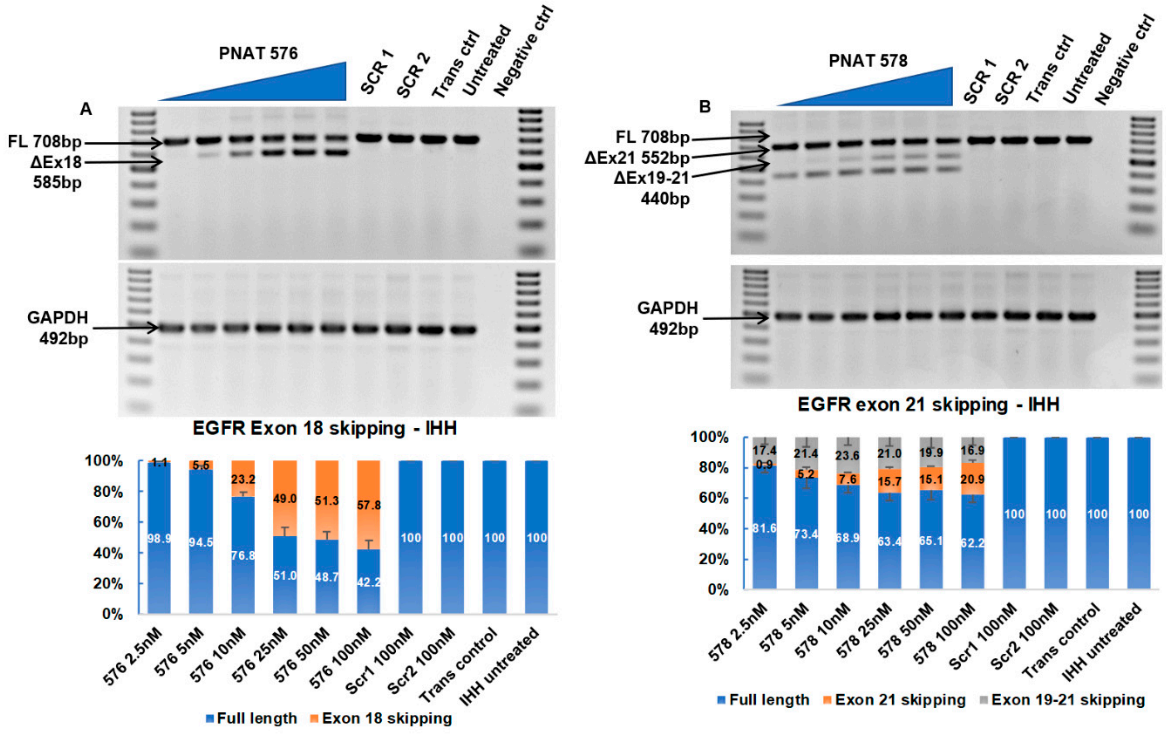

Figure S13: RT-PCR and densitometry analysis of exon 18 and 21 skipping in IHH cells. (A) Agarose gel showing dose-dependent exon 18 skipping following PNAT576 transfection in IHH cells and percentage exon 18 skipping assessed by densitometry (B) Agarose gel showing dose-dependent exon 21 and partial 19-21 skipping following PNAT578 transfection in IHH cells and percentage exon 21 and partial 19-21 skipping assessed by densitometry. The dose concentration was 2.5, 5, 10, 25, 50 and 100nM. The scrambled sequences SCR1 and SCR2 were transfected at 100nM concentration. Densitometry was performed for the biological triplicates and the exon skipped and full-length bands were normalised to respective GAPDH first and then to untreated sample. The data are represented as mean±SEM.

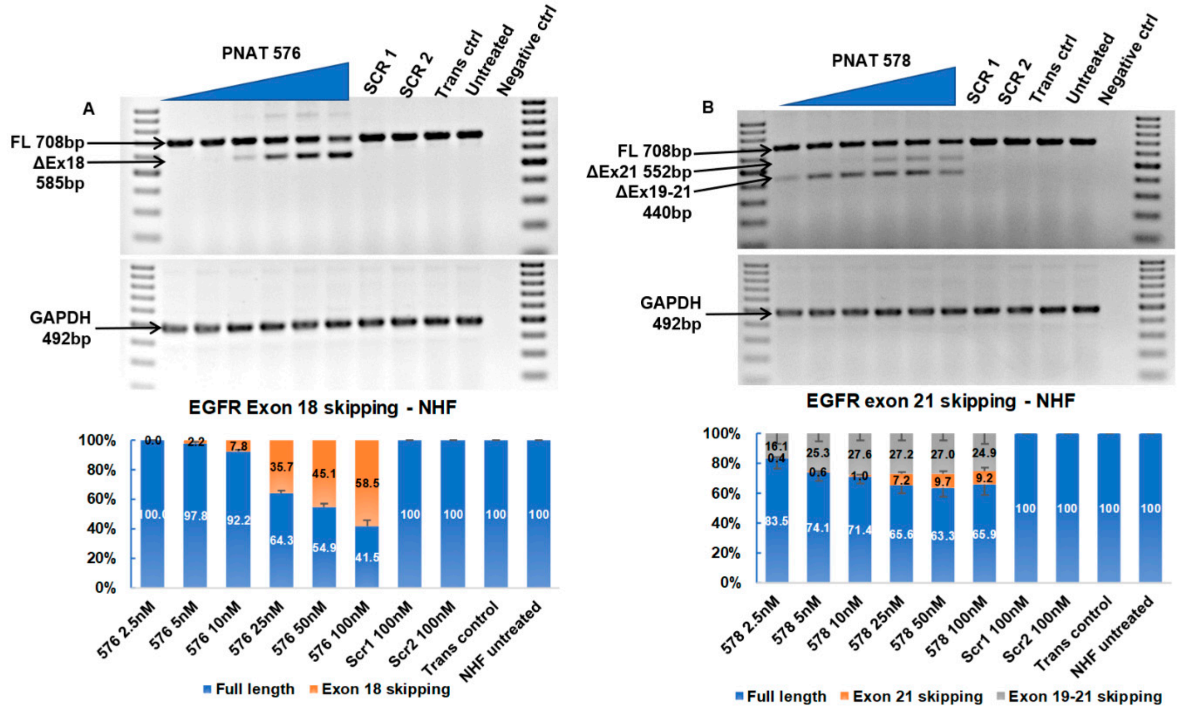

Figure S14: RT-PCR and densitometry analysis of exon 18 and 21 skipping in NHF cells. (A) Agarose gel showing dose-dependent exon 18 skipping following PNAT576 transfection in NHF cells and percentage exon 18 skipping assessed by densitometry (B) Agarose gel showing dose-dependent exon 21 and partial 19-21 skipping following PNAT578 transfection in NHF cells and percentage exon 21 and partial 19-21 skipping assessed by densitometry. The dose concentration was 2.5, 5, 10, 25, 50 and 100nM. The scrambled sequences SCR1 and SCR2 were transfected at 100nM concentration. Densitometry was performed for the biological triplicates and the exon skipped and full-length bands were normalised to respective GAPDH first and then to untreated sample. The data are represented as mean±SEM.

Table S1: Primers for EGFR and GAPDH amplification

Primer name	Sequence 5' – 3'	Amplicon size
hEGFR_1 Ex1F	CGGGCTCTGGAGGAAAAGAAAG	457bp
hEGFR_1 Ex4R	TGCTGAGAAAGTCACTGCTGA	
hEGFR_4 Ex17F	GATCGGCCTCTTCATGCGAA	708bp
hEGFR_4 Ex22R	CTCCAGACATCACTCTGGTGG	
GAPDH-fwd	GGACTCATGACCACAGTCCATGC	492bp
GAPDH-rev	TTACTCCTTGGAGGCCATGTGGG	

Table S2: PCR conditions for *EGFR* exon 1-4 amplification.

Temp	Time	Cycles
55℃	30 mins	
94℃	2 mins	
94℃	30 s	30
55℃	1 min	
68℃	2 mins	

Table S3: PCR conditions for *EGFR* exon 17-22 amplification.

Temp	Time	Cycles
55℃	30 mins	
94℃	2 mins	
94℃	30 s	28
55℃	1 min	
68℃	2 mins	

Table S4: PCR conditions for *GAPDH* amplification.

Temp	Time	Cycles
55℃	30 mins	
94℃	2 mins	
94℃	30 s	25
60℃	1 min	
68℃	2 mins	
